# Chinese Medicinal Leech: Ethnopharmacology, Phytochemistry, and Pharmacological Activities

**DOI:** 10.1155/2016/7895935

**Published:** 2016-05-04

**Authors:** Han Dong, Ji-Xiang Ren, Jing-Jing Wang, Li-Shuai Ding, Jian-Jun Zhao, Song-Yan Liu, Hui-Min Gao

**Affiliations:** ^1^Department of Neurology, China-Japan Union Hospital, Jilin University, Changchun 130033, China; ^2^The Affiliated Hospital to Changchun University of Chinese Medicine, Changchun 130021, China; ^3^Institute of Chinese Materia Medica, China Academy of Chinese Medical Sciences, Beijing 100700, China; ^4^National Engineering Laboratory for Quality Control Technology of Chinese Herbal Medicine, Beijing 100700, China; ^5^College of Pharmacy, Henan University of Traditional Chinese Medicine, Zhengzhou 450008, China

## Abstract

Hirudo (Shuizhi in Chinese) is an important Chinese medicine, which possesses many therapeutic properties for the treatment of the cerebral hemorrhage and other thrombosis-related diseases. The phytochemical investigation gave more than 51 compounds including pteridines, phosphatidylcholines, glycosphingolipids, and sterols, as well as some bioactive peptides from the Shuizhi derived from three animal species recorded in the current Chinese Pharmacopoeia. The pharmacological studies on the Shuizhi have revealed various activities such as anticoagulation, antithrombosis, antiatherosclerosis, antiplatelet aggregation, antitumor and anti-inflammatory as well as hemorheology improvement, and protective effects against cerebral ischemia-reperfusion injury. However, some important issues based on the traditional uses of Shuizhi are still not clear. The aim of the present review is to provide comprehensive knowledge on the ethnopharmacology, phytochemistry, and pharmacological activities of Shuizhi. It will provide a potential guidance in exploring main active compounds of Shuizhi and interpreting the action mechanism for the further research.

## 1. Introduction

Cardiovascular diseases (CDs) are the leading cause of death worldwide [1]. They resulted in over 17.3 million deaths (31.5%) in 2013 [2]. The pathological thrombus formation is responsible for most of CDs such as myocardial infarction and strokes. Compared with the synthetic drugs, Traditional Chinese Medicines (TCMs) have been evidenced to be active in the prevention and treatment of thrombosis-related diseases with few side effects. Hirudo, a representative of animal-sourced TCMs, also known as Chinese medicinal leech (Shuizhi in Chinese), is featured on promoting blood circulation and removing blood stasis. It was firstly recorded in the* Shen-Nong-Ben-Cao-Jing* and had been widely used for the treatment of cardiovascular diseases and other chronic diseases [3]. More than 300 prescriptions containing Shuizhi have been used in the clinical practice for 2000 years [4] and there are hundreds of manufacturers making single Shuizhi or its formula preparations in China [5]. Since 1963, Shuizhi has been listed in the Chinese Pharmacopoeia [6].

Hirudotherapy was once popular in Europe, especially during the 1825 and 1850, owing to the excluded effect on treating various human diseases [7]. For several years, there has been renewed interest in the use of medicinal leeches, especially for reducing blood coagulation, relieving venous pressure from pooling blood, and treating congestive complications after plastic and reconstructive surgery [8, 9]. Widespread recognition and acceptance of leech usage within the medical community came in 2004 when the US Food and Drug Administration officially approved the marketing and sale of the medicinal leech as a medical device for relieving venous congestion [10, 11]. 650 species of leeches are found all over the world and* Hirudo medicinalis* is a kind of leech medically used in the western countries [9]. However, according to the description in the Chinese traditional literatures, only several species, with the features of small body, living in water, and blood-sucking, are possible sources of Shuizhi, such as* H*.* nipponia* Whitman (HN),* H*.* pulchra* Song,* Poecilobdella nanjingensis* sp. Nov.,* P*.* manillensis* (Lesson), and* P*.* hubeiensis* Yang [12]. Three species including* Whitmania pigra* Whitman (WP),* H*.* nipponica* Whitman, and* W*.* acranulata* Whitman (WA) are recorded in the current Chinese Pharmacopoeia as the source of Shuizhi [13]. Among them, WP is the most commonly available from Chinese commercial leech market. Based on the potent anticoagulant effect of Shuizhi, many investigations on phytochemistry and biological activities have been reported. The present review summarized the ethnopharmacology, phytochemistry, and pharmacological properties of Shuizhi, concerning three legally listed Chinese species, which can provide some evidences for the interpretation of its effective constituents and action mechanism.

## 2. Ethnopharmacology 

The earliest record of Shuizhi appeared in the first classic book on Chinese Materia Medica,* Shen-Nong-Ben-Cao-Jing* as a downgrade drug. The debate on the animal origins of Shuizhi has been continued in the follow-up Chinese medical classics* Ming-Yi-Bie-Lu* (206 BC–8 AD),* Ben-Cao-Jing-Ji-Zhu* (480–498 AD),* Xin-Xiu-Ben-Cao* (657–659 AD),* Shu-Ben-Cao* (935–960 AD),* Ben-Cao-Tu-Jing* (1020–1101 AD),* Ben-Cao-Meng-Quan* (1565 AD), and* Ben-Cao-Gang-Mu* (1590 AD). As an important TCM characterized by eliminating blood stasis and stimulating menstruation discharge, the Chinese Pharmacopoeia has officially accepted it since 1963, for the treatment of amenorrhea induced by blood stasis, masses in the abdomen, apoplexy, and hemiplegic paralysis as well as traumatic injuries.

Shuizhi is often used in combination with other TCM ingredients to treat ailments resulting from blood stasis. Based on the textual literatures, more than 300 prescriptions containing Shuizhi have been described in the clinical practice, including the different dosage forms such as pill, decoction, powder, sublimed preparation, and plaster. The famous “Di-Dang decoction or pill” and “Da-Huang-Zhe-Chong pill” are well employed by medical sage, Zhang Zhong-jing, living in the Eastern Han Dynasty (25–220 AD). In the mentioned prescriptions, there are up to 290 TCMs being compatible with Shuizhi, among which, the top ten drugs are* Tabanus* (Mengchong),* Persicae Semen* (Taoren),* Rhei Radix et Rhizoma* (Dahuang),* Angelicae Sinensis Radix* (Danggui),* Cinnamomi Cortex* (Rougui),* Moutan Cortex* (Mudanpi),* Achyranthis Bidentatae Radix* (Niuxi),* Ginseng Radix et Rhizoma* (Renshen),* Natrii Sulfas* (Mangxiao), and* Toxicodendri Resina* (Ganqi) (Table S1, in Supplementary Material available online at http://dx.doi.org/10.1155/2016/7895935). The herbal couple of* Hirudo* (Shuizhi) and* Tabanus* (Mengchong) is the most common unit in the prescription compositions.

According to recorded formula compositions and clinical evidences, most of these prescriptions have been subjected to modern drug research and development. As a result, to date, there are hundreds of patented preparations officially approved to the medical market in China, containing single Shuizhi or its compatibility form with other herbs (Table S2) [5, 13, 14]. The representatives of frequently prescribed preparations are Nao-Xue-Kang, Huo-Xue-Tong-Mai Capsule, and Tong-Xin-Luo Capsule. The former two are derived from single Shuizhi and the latter is prepared from eleven ingredients:* Ginseng Radix et Rhizoma* (Renshen),* Hirudo* (Shuizhi),* Scorpio* (Quanxie),* Paeoniae Radix Rubra* (Chishao),* Cicadae Periostracum* (Chantui),* Eupolyphaga* (Tubiechong),* Scolopendra* (Wugong),* Santali Albi Lignum* (Tanxiang),* Dalbergiae Odoriferae Lignum* (Jiangxiang),* Olibanum* (Ruxiang, processed with vinegar),* Ziziphi Spinosae Semen* (Suanzaoren, fried), and* Borneolum Syntheticum* (Bingpian).

## 3. Chemical Constituents

The enormous information gathered from the ethnopharmacological applications of Shuizhi and its preparations needs the global investigation of the chemical constituents. These studies have led to the isolation of macromolecule substances such as protein and polypeptide and small molecules including pteridines, phosphatidylcholines, glycosphingolipids, and sterols.

### 3.1. Proteins and Peptides

#### 3.1.1. Hirudin and Recombinant Products

As the representative of active proteins and peptides from Shuizhi, hirudin has been considered as the most potent natural inhibitor of thrombin for a long time. Hirudin is an active peptide isolated from the saliva of* H*.* medicinalis* by Marquanrdt of Germany in 1950 and it is composed of 65 amino acids with the molecular weight of approximately 7.1 kDa, including a compact* N*-terminal domain containing three S-S bonds and a* C*-terminal domain that is disordered in uncomplexed hirudin [15]. Natural hirudin presents in minuscule amounts, and it is not enough to obtain hirudin as an antithrombotic agent for considerable clinical use. Therefore, recombinant techniques are used to produce homogeneous preparations of hirudin. In a successful case, the recombinant RGD-hirudin containing a recognizable sequence of Arg-Gly-Asp (RGD) is a bifunctional molecule according to the structure of wild-type hirudin variant 2. These structural changes improve the hydrophobicity of the protein and allow the recombinant RGD-hirudin to interact more effectively with the fibrinogen recognition exosite of thrombin, resulting in a specific activity of 12,000 ATU/mg [16]. Owing to the significant anticoagulant effects [17, 18], recombinant hirudin has been approved into the market for the treatment of thrombosis-related diseases.

Hirudin naturally occurs in the fresh saliva of medicinal leeches, and it is almost not detected in the processed Shuizhi samples. Moreover, hirudin is easily degraded when orally administrated by the pepsin secreted from gastric chief cells. However, according to the traditional uses, Shuizhi was commonly decocted in hot water for 1-2 h and orally administrated in the clinical practice, resulting in the good anticoagulant effect [19]. The evidences from the comparative study of different extracting methods also suggested the extracts produced by water-boiling and alcohol-precipitating showed the strongest anticoagulant activity and good antithrombotic effect [20]. These clues led to more investigations for discovering other potent substances from Shuizhi.

#### 3.1.2. Other Peptides

Recent studies reported novel proteins or peptides with anticoagulant activity from Shuizhi related animal origin species. NLP-1 (New Leech Protein-1), a low-abundant protein (Mw 13800 Da), was obtained from HN on the basis of biomimetic ligand library screening and one-step purification method [21]. Recently, three peptides were also identified from dried body of HN with their molecular weight separately 14998, 15988, and 15956 Da by ultra-performance liquid chromatography coupled with electrospray ionization quadrupole time-of-flight mass spectrometric detector [22]. Using the same methods, an oligopeptide with a much lower molecular weight of 1997.1 Da, whitide, was obtained from dried WP, and it might be an oral anticoagulant for its hot and trypsin stability [23]. A novel anticoagulant peptide, whitmanin, was isolated and purified from the dried body of WP by anion-exchange chromatography on Sephadex DEAE A-50, gel permeation chromatography on Sephadex G-25 and Sephadex LH-20 columns, and reversed phase high performance liquid chromatography successively [24, 25].

### 3.2. Small Molecule Constituents

Apart from the macromolecules, small molecule constituents are considered as potent active substances, including pteridines, phosphatidylcholines, glycosphingolipids, sterols, and fat acids.

#### 3.2.1. Pteridines

**Figure 1:**
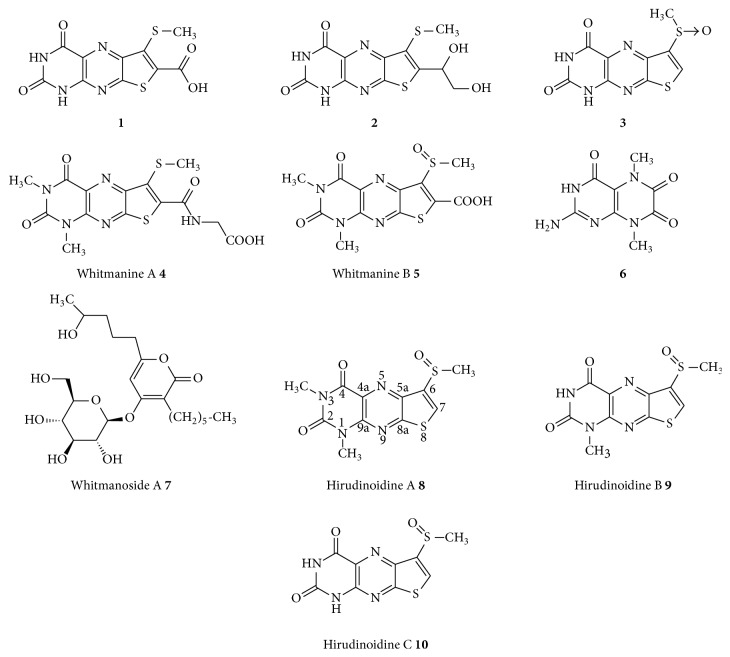
Structures of the pteridine derivatives isolated from Shuizhi.

Two new heterocyclics, hirudonucleodisulfide A** 1** and hirudonucleodisulfide B** 2**, were isolated from the dried material of WP, displaying the moderate antianoxic activity with EC_50_ values of 27.01 ± 2.23 *μ*g/mL and 19.54 ± 1.53 *μ*g/mL, respectively [26]. The same authors' continual investigation on WP reports the isolation and structural elucidation of a new pteridinedione** 3** [27]. From the same species, three new pteridine derivatives,** 4**–**6**, and a new *α*-pyrone glycoside** 7**, with novel structural features, were publicated recently [28, 29]. Interestingly, the phytochemical study on HN, another legally listed species, also afforded three new pteridines, hirudinoidines A** 8**, B** 9**, and C** 10** [30], which suggested both species shared the same compound type. The structures of compounds** 1**–**10** are presented in Figure 1.

#### 3.2.2. Phosphatidylcholines and Glycosphingolipids

**Figure 2:**
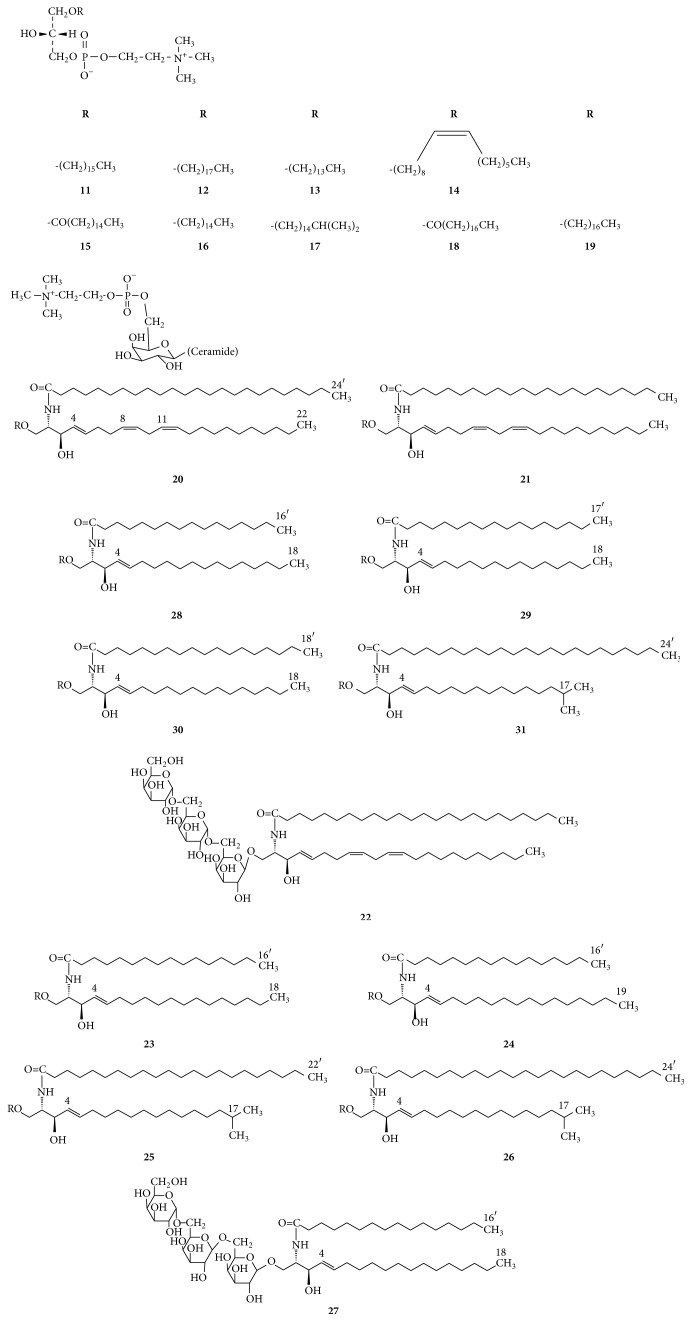
Structures of phosphatidylcholines and glycosphingolipids.

Nine lysoglycerophospholipids** 11**–**19** were isolated from the dried body of HN [31]. Among them, two (**15** and** 18**) are lysophosphatidylcholines and the other seven are lyso-platelet-activating factors. Eight new glycosphingolipids** 20**–**27**, along with four known ones,** 28**–**31**, were also obtained from this species, including six zwitterionic monogalactosylceramides carrying a choline phosphate group,** 20**-**21** and** 28**–**31**, and six neutral trigalactosylceramides** 22**–**27** [32, 33]. The structures of compounds** 11**–**31** are presented in Figure 2.

#### 3.2.3. Other Components

Eight known compounds have been reported for the first time from HN collected from Jiangsu province, China, including campesterol** 32**, hexadecyl ether of glycerol** 33**, (2*S*,3*S*,4*E*)-4,5-didehydrooctadecasphingosine pentacosanoic amide** 34**, 1-*O*-(*β*-*D*-galactopyranosyloxy)-substituted-2-(docosenoylamino)octadec-4-en-3-ol** 35**, succinic acid** 36**, hypoxanthine** 37**, propylamine** 38,** and L-isoleucine** 39** [34]. In addition to the compounds** 32**,** 33**, and** 36**–**39**, other 12 first-reported constituents are obtained from commercial samples of HN, including cholesterol** 40**, nicotinic acid** 41**, uracil** 42**, uridine** 43**, inosine** 44**, xanthine** 45**, phenylalanine** 46**, adenosine** 47**, proline** 48**, valine** 49**, glycerin** 50**, and palmitic acid** 51** [35]. Fatty acid methyl esters and sterols were identified by GC-MS in the anticoagulant extract of WP [36].

## 4. Qualitative and Quantitative Analyses

Since the protein and amino acids are the major components of Shuizhi, the amino acids targeted analysis on Shuizhi is one of the main aspects in the quality control. A reversed phase high performance liquid chromatography-evaporative light scattering detection method was developed for the direct determination of fourteen underivatized amino acids in the samples of WP, including serine, histidine, glycine, threonine, arginine, alanine, tyrosine, tryptophan, methionine, valine, phenylalanine, isoleucine, leucine, and lysine [37]. Compared with direct determination method without derivatization, the indirect methods with derivatization of amino acid (pre- or post-column derivatization) were commonly used for the total amino acids and free amino acids in the dried Shuizhi samples [38, 39]. For the small bionic zymolysis peptides, Lorry method recorded in the Chinese Pharmacopoeia was more preferable than biuret method and ninhydrin colorimetry method [40, 41]. Dot blotting was also employed for the determination of hirudin's hydrolysates in the processed Shuizhi samples with rat antibody of anti-hirudin as the first antibody, resulting in the concentration of 296.51, 165.47, 95.58, and 298.05 *μ*g/g [42]. The new anticoagulant peptide, whitmanin, has the average concentration of 0.074% in the different batches of commercial samples from WP [43].

Nucleosides have been proven as the bioactive compounds involved in the multiple biological activities such as antiplatelet aggregation and antiarrhythmic and antiseizure effects [44]. Several quantitative studies have been reported concerning the recently reported small molecule compounds as marker constituents for the quality evaluation of Shuizhi samples, including uracil, hypoxanthine, xanthine, uridine, hirudonucleodisulfide C, hirudonucleodisulfide A, and hirudonucleodisulfide B [45–50]. The content of uracil, hypoxanthine, xanthine, uridine, hirudonucleodisulfide C, hirudonucleodisulfide A, and hirudonucleodisulfide B in the different batches of samples varies over the range of 0.063–0.17%, 0.90–1.48%, 0.052–0.23%, 0.051–0.17%, 0.18–0.28%, 0.15–0.29%, and 0.12–.021%, respectively. The total amounts are 2.12–2.41% [48]. The content of hirudonucleodisulfide C was the highest and that of hirudonucleodisulfide B was the lowest. In addition, the total amounts of three compounds depended on the species and cultivating areas [49].

In terms of the ongoing debate on the undefined effective substances of Shuizhi, the quality control using the chemical markers displayed certain limitation. Therefore, the bioassay has been paid more attention to. Apart from the items including description, TLC identification, tests (water content, total ash, ash insoluble in hydrochloric acid, and pH value), and ethanol soluble extractive, the antithrombin titration method for the quantitative assay was legally recorded in the current quality standard of Shuizhi [13]. It contains not less than 16.0 U of antithrombin per g for HN and 3.0 U for WP and WA. However, this quantitative method is not so objective and it could lead to poor accuracy of the test results. Many methods based on the biological activity were attempted to improve the quality control strategy of Shuizhi, such as fibrinogen-thrombin time (Fibg-TT) [51] and activated partial thromboplastin time (APTT) [52–54].

## 5. Pharmacological Activities

As an important TCM to control cerebral hemorrhage and other thrombosis-related diseases, Shuizhi showed various pharmacological effects including anticoagulation, antithrombosis, antiatherosclerosis, antiplatelet aggregation, antitumor and anti-inflammatory as well as hemorheology improvement, and protective effects against cerebral ischemia-reperfusion injury. Of all the activities described for Shuizhi, the anticoagulation and antithrombosis are the most widely studied subject to date. In terms of traditional uses, we provide a general overview of main bioactivity of Shuizhi extract, powder, or micropowder. The progress on the pharmacological activity of hirudin is beyond the scope of this paper.

### 5.1. Effect on the Hematological System

#### 5.1.1. Anticoagulation

Numerous studies have evidenced that the anticoagulation is the main pharmaceutical effect of Shuizhi [55]. However, the action intensity of anticoagulation is affected by many factors such as origin species [56–58], harvesting and postharvesting process [59–61], and preparation methods of tested samples [62, 63]. The Shuizhi samples from blood-sucking HN displayed stronger anticoagulation activity than those from snail or clam-feeding WP, and, moreover, the fresh samples are more active than the dried ones [56]. The extracts obtained by different extract solvents and methods showed various anticoagulant activities. Prothrombin time (PT), thrombin time (TT), and APTT were employed as evaluation markers for the discovery of active fractions of Shuizhi and, as a result, ethyl acetate fraction remarkably prolonged PT, TT, and APTT [62]. Nonheating leech extract could prolong bleeding time (BT) and clotting time (CT) in the model mice and PT and APTT in the model rats and decrease the activity of coagulation factor II and platelet aggregation in the model rats [64]. Nonheating leech extract produced stronger anticoagulant effect on the mice with blood hypercoagulable state than water-decocted leech extract [65]. Altogether, the different species, habitat, harvest time, extraction methods, and drying process would result in the different quality of Shuizhi with variable anticoagulant activity.

Most of published literatures report that the anticoagulation mechanism of Shuizhi is related to hirudin. However, hirudin level is very low in the processed Shuizhi samples, even almost not detected. The increasing evidence indicates that other small molecules may be attributed to the whole anticoagulation effect. Therefore, more efforts are to be made for understanding its action mechanism.

#### 5.1.2. Antithrombosis Effect

The antithrombosis effect shares part of the endogenous or exogenous signal pathways with anticoagulation. Besides anticoagulation, Shuizhi showed potent antithrombosis effect. The ethanol extract from Shuizhi significantly inhibited the thrombosis induced by collagen-adrenaline in the mice and thrombosis in artery-vein bypass in the rats. This effect may be associated with the enhancement of erythrocyte and thrombocyte membrane fluidity [66, 67]. The water-soluble extract of Shuizhi significantly activated tissue factor pathway inhibitors and inhibited overexpression of tissue factors in the HUVECs induced by thrombin [68].

#### 5.1.3. Effect on Hemorheology

Shuizhi water-decocted extract can decrease blood viscosity and plasma viscosity of rats or human and shorten the electrophoresis time of erythrocyte [69]. The active extracts of Shuizhi can significantly improve hemorheological parameters of the acute blood stasis rats [70]. Shuizhi micropowder can improve blood rheology of rats with acute blood stasis and possess obvious effect of promoting blood circulation and removing blood stasis. Its action mechanism might be related to the content decrease of plasma von Willebrand factor and plasminogen activator inhibitor-1 [71].

#### 5.1.4. Antiatherosclerosis Effect

Atherosclerosis is a main event and fundament of many cardiovascular diseases and it has now been considered as a chronic inflammatory disease. Shuizhi extract from WP can obviously attenuate the area of atherosclerosis lesion in ApoE−/− mice in a dose-dependent manner, and this effect is mainly a result of reduced invasion of monocyte in artery walls by blocking NF-*κ*B translocation [72]. Shuizhi powder may be involved in the decrease of plasma total cholesterol level and inflammatory factor, further inhibition on the proliferation of smooth muscle cells in the atherosclerotic lesions in apolipoprotein E deficient mice [73]. Shuizhi extract at the dosage of 3, 15, and 75 mg/kg decreased significantly the content of plasma total cholesterol, triglyceride, low density lipoprotein-cholesterol and nitric oxide, and activities of serum NOS and iNOS and increased activities of serum cNOS in hyperlipidemia rats [74]. An experiment carried out in blood stasis syndrome rabbits further confirmed that Shuizhi could regulate lipid metabolism, which is related to increasing LDL-R and ApoE mRNA expression [75]. Liu and Sui have drawn similar conclusion that Shuizhi superfine powder played a role in antagonizing atherosclerosis by regulating level of serum lipid [76]. Tongxinluo capsule, a compound preparation containing Shuizhi as a main ingredient, has similar effects as simvastatin in lowering serum lipid levels, inhibiting plaque inflammation, and preventing vulnerable plaques from rupture and may provide an alternative therapy for atherosclerosis [77]. Dahuang Zhechong pill, a famous and classical Chinese herbal prescription containing Shuizhi, significantly inhibited the proliferation of vascular smooth muscle cells (VSMCs) in vivo and in vitro. The inhibitory effect is partially attributed to depressing PDGF expression in VSMCs, retarding the cell cycle and promoting apoptosis of VSMCs [78]. Reversion of vascular endothelial dysfunction is another important mechanism of antiatherosclerotic effect of Shuizhi [79].

### 5.2. Protective Effect against Cerebral Ischemia-Reperfusion Injury

As an important ingredient in the clinical prescriptions for cerebral hemorrhage, anticoagulation cannot fully explain the efficacy of Shuizhi. The cerebral ischemia-reperfusion injury was characterized by the injury, apoptosis, and necrosis of nerve cells. The Shuizhi extract obtained by water decoction and ethanol precipitation significantly protected cerebral cells from apoptosis in middle cerebral artery ischemia/reperfusion rats by reducing the apoptotic rate of cerebral cells [80] and regulation of Bcl-2 and Bax protein [81]. Shuizhi micropowder also displayed the protective effect on cerebral ischemia-reperfusion injury by increasing SOD activity and decreasing MDA and NO contents in the serum or cerebral tissues, as well as reducing the production of inflammatory factors such as intercellular adhesion molecule-1 (ICAM-1), vascular cell adhesion molecule-1 (VCAM-1), and platelet-derived growth factor (PDGF) [82–84]. Similar mechanism was shown that Sheng-Nao-Kang decoction (SNK) including Shuizhi as one of fifteen TCM ingredients, a modified traditional Chinese medicine for the treatment of acute and chronic cerebrovascular related diseases, demonstrated a strong and ameliorative effect on cerebral I/R damage in rats due to the properties of antiapoptosis and antioxidation as well as regulation of iNOS and TNOS [85]. In addition, the effect of Shuizhi on cerebral hemorrhage might be associated with the production improvement of capillary cell and glial cells in rats [86]. Under the guidance of “Nao sui” theory proposed by Ren Jixue, National Master of TCM, Poxue Huayu and Tianjing Busui decoction, containing Shuizhi in combination with other seven TCMs, was used for cerebral hemorrhage. Its action mechanism is through upregulating the expression of brain-derived neurotrophic factor [87].

### 5.3. Antitumor Effect

Shuizhi is the representative of TCMs derived from animal insect sources, with the properties of eliminating blood stasis, softening hard masses, and dissolving lumps. The single Shuizhi or its formula preparations in combination with other TCM ingredients were commonly used to treat various cancers in the clinical practice, including esophagus cancer, gastric carcinoma, intestinal cancer, hysterocarcinoma, and breast cancer. The increasing evidence indicated that Shuizhi could inhibit the proliferation of human HepG2 [88, 89] and leukemic HL-60 cells [90]. The action mechanism could be involved in the induced cell differentiation and apoptosis [88, 90, 91] and cell cycle arrest in the G1 phase [92], as well as participation in DNA demethylation by inhibiting the expression of DNA methyltransfer [89, 93]. Downregulating the expression of MDR1 and upregulating caspase 3 are considered to be possible mechanism of Shuizhi extract inducing apoptosis and enhancing the chemotherapeutic sensitivity of 5-fluorouracil and adriamycin in the Hep2 cell [94]. In addition, a recent investigation suggested that Shuizhi could inhibit tumor angiogenesis via improving tumor hypoxia microenvironment, which was partly attributed to decreasing the expression of mRNA and protein level of hypoxia inducible factor-1*α* (HIF-1*α*), and degrade the mRNA expression of vascular endothelial growth factor (VEGF), downstream gene of HIF-1*α* [95, 96].

### 5.4. Anti-Inflammatory Effect

Shuizhi has displayed anticoagulant, antithrombin, and hypolipidemic effect as well as protective effect against cerebral ischemia-reperfusion injury. These effects could be attributed to its anti-inflammation and detumescence. Earlier reports indicated that Shuizhi extract from WP at a dose of 2 g crude material/kg reduced abdominal capillary permeability in the mice and relieved croton oil-induced mouse ear edema and carrageenan-induced rat paw edema and subcutaneous embedding of filter paper induced granulation tissue hyperplasia in the mice [97]. The processed products showed a stronger anti-inflammatory trend than that of crude materials [98].

## 6. Concluding Remarks and Future Perspectives

As a valuable traditional medicine, the research on chemical compositions, pharmacological actions, and therapeutic properties of Shuizhi has been ongoing in China, and, up to now, great progress has been made; however, its active substances and action mechanisms remain unclear.

### 6.1. Influence of Origin Species on the Chemical Difference and Therapeutic Properties of Shuizhi

Of all three species legally described in the Chinese Pharmacopoeia, WP is the richest resource and it has become mainstream in the Chinese commercial Shuizhi market. However, compared with blood-sucking HN, WP showed weaker anticoagulation effect and, moreover, it is not in accordance with the description of medicinal leech in the Chinese traditional textures [12]. For the multiorigin TCMs, each of different species displayed various pharmacological intensity, which is bound to bring to the effectiveness difference in the clinical practice. Therefore, the comprehensively systematic comparison of the chemistry and pharmacology of three legally recorded species is necessary for the consistency of clinical efficacy of commercially available Shuizhi.

### 6.2. The Debate on the Active Substances Is Ongoing and Much Effort Is to Be Made

Most studies regarded hirudin and its homologue peptides as active substances responsible for its anticoagulation activity; however, in our opinion, the following points support that more components are attributed to the whole effect of Shuizhi. Hirudin is present in the fresh saliva of HN and it is easily destroyed at high temperature or by the pepsin. It is active in the direct injection form. Traditionally, Shuizhi was commonly used in the processed forms (calcining in the hot talcum powder) and decocted in the hot water before being orally administrated. Therefore, based on the ancient tradition, it is not enough to take hirudin as active substances for Shuizhi samples. The studies on the active substances of Shuizhi and their action mechanism are to be continued, employing the advanced extraction, purification, and analytical technologies, including proteomics for macromolecules and phytochemistry for micromolecules as well as the integration of metabonomics, network pharmacology, and chemoinformatics.

### 6.3. The Art and Science of Processing for Shuizhi in the Clinical Practice

According to traditional Chinese medicine theory, paozhi processing transforms raw drugs into “decoction pieces,” thus instilling them with the desired properties for their medical application, including improved flavor and detoxification or alteration of their therapeutic efficacy [99]. Based on the ancient tradition, it is necessary for Shuizhi to be processed before prescribing, by calcining, or frying with or without adjuvants. In the current Chinese Pharmacopoeia, the only calcined Shuizhi in the hot talcum powder is recorded. The studies indicated that processing at the high temperature could decrease the anticoagulation activity of Shuizhi or improve its anti-inflammatory activity [100]. How to obtain the targeted therapeutic features by processing is very important, which is associated with the different factors such as tested samples, processing parameters, and bioassay markers. More investigations are to be done for understanding the scientific importance of processing of Shuizhi for the clinical prescription and drug preparations and further standardization of the processing techniques.

Generally speaking, the present review summarized the traditional uses and phytochemistry and pharmacological properties of Shuizhi. Some important unanswered questions and directions for future research are outlined, which arouses us to take more efforts to assure the safety and efficacy of Shuizhi in the clinical practice.

## Supplementary Material

Chinese traditional uses of Shuizhi and its formula prescriptions were shown in Table S1. The patented drugs containing Shuizhi and their clinical uses (222 Drug approval number) were shown in Table S2.
