# Supplementary material for: Chinese Medicinal Leech: Ethnopharmacology, Phytochemistry, and Pharmacological Activities
Source: Evid Based Complement Alternat Med. 2016 May 4;2016:7895935. doi: 10.1155/2016/7895935 (PMC4870366; doi:10.1155/2016/7895935)
Supplement: Supplementary file 1 — Chinese traditional uses of Shuizhi and its formula prescriptions were shown in Table S1. The patented drugs containing Shuizhi and their clinical uses (222 Drug approval number) were shown in Table S2. [file 7895935.f1.pdf]

Chinese Medicinal Leech: Ethnopharmacology, Phytochemistry and Pharmacological Activities

Han Dong [1], Ji-Xiang Ren [2], Jing-Jing Wang [3,4], Li-Shuai Ding [3,4], Jian-Jun Zhao [2], Song-Yan Liu [4]*, Hui-Min Gao [3,]* , Zhi-Min Wang [3]

1 Department of Neurology, China-Japan Union Hospital, Jilin University, Changchun, 130033, China; E-Mail: 441652741@qq.com (H. Dong); E-Mail: yan1966@163.com (S.-Y. Liu)

2 The Affiliated Hospital to Changchun University of Chinese Medicine, Changchun 130021, China; E-Mails: renjx2003@163.com (J.-X. Ren); zhaojianjun8777@163.com (J.-J. Zhao)

3 Institute of Chinese Materia Medica, China Academy of Chinese Medical Sciences, Beijing 100700, PR China; National Engineering Laboratory for Quality Control Technology of Chinese Herbal Medicine, Beijing 100700, PR China; E-Mails: jingwqt@163.com (J.-J. Wang); 990698360@qq.com (L.-S. Ding); huimin_gao@126.com (H.-M. Gao); zhmw123@263.net (Z.-M. Wang).

4 College of Pharmacy, Henan University of Traditional Chinese Medicine, Zhengzhou 450008, China; E-Mails: jingwqt@163.com (J.-J. Wang); 990698360@qq.com (L.-S. Ding).

* Author to whom correspondence should be addressed; E-Mail: yan1966@163.com (S.-Y. Liu); huimin_gao@126.com (H.-M. Gao), Tel.: +86-10-84014128; Fax: +86-10-84014128.

Table S1 Chinese traditional uses of Shuizhi and its formula prescriptions

**Formulae**	**Main compositions**	**Clinical uses**	**References**
Baiwei Wan	*Cynanchi atrati radix et rhizoma* (Baiwei), *Asari radix et rhizoma* (Xixin), *Magnoliae officinalis cortex* (Houpo), *Zanthoxylum bungeanum pericarpium* (Huajiao), *Platycodonis radix* (Jiegeng), *Trionycis carapax* (Biejia), *Saposhnikoviae radix* (Fangfeng), *Rhei radix et rhizoma* (Dahuang), *Aconiti lateralis radix praeparata* (Fuzi), *Sulfur* (Liuhuang), *Rubia yunnanensis* Diels (Zishen), *Ginseng radix et rhizoma* (Renshen), *Taxilli herba* (Sangjisheng), *Pinelliae rhizoma* (Banxia), *Bombyx batryticatus* (Jiangchan), *Dipsaci radix* (Xuduan), *Gentianae macrophyllae radix* (Qinjiao), *Asteris radix et rhizoma* (Ziwan), *Achyranthis bidentatae radix* (Niuxi), *Tabanus* (Mengchong), ***Hirudo*** (Shuizhi), *Fluoritum* (Zishiying), *Natrii Sulfas* (Mangxiao), *Cinnamomi cortex* (Rougui), *Stalactitum* (Zongrushi), *Angelicae sinensis radix* (Danggui).	Regulating the menstrual function. Treating female infertility	You-you-xin-shu
Bailao Wan	*Angelicae sinensis radix* (Danggui), *Olibanum* (Ruxiang), *Myrrha* (Moyao), *Tabanus* (Mengchong), *Rhei radix et rhizoma* (Dahuang), *Ginseng radix et rhizoma* (Renshen), ***Hirudo*** (Shuizhi), *Persicae semen* (Taoren).	Regulating the menstrual function	Jiang-xue-yuan-Gu-fang-xuan-zhu
Banmao Wan	*Mylabris* (Banmao), *Moschus* (Shexiang), ***Hirudo*** (Shuihzi), *Glycyrrhizae radix et rhizoma* (Gancao), *Sojae semen nigrum* (Heidou), *Rhei radix et rhizoma* (Dahuang), *Zaocys* (Wushaoshe).	Crewels and carbuncle	Tai-ping-sheng-hui-Fang
Biejia Wan	*Trionycis carapax* (Biejia), *Cinnamomi cortex* (Rougui), *Vespae Nidus* (Fengfang), *Scrophulariae radix* (Xuanshen), *Zanthoxylum bungeanum pericarpium* (Huajiao), *Asari radix et rhizoma* (Xixin), *Ginseng radix et rhizoma* (Renshen), *Sophorae flavescentis radix* (Kushen), *Salviae miltiorrhizae radix et rhizoma* (Danshen), *Adenophorae radix* (Nanshashen), *Euodiae Fructus* (Wuzhuyu), *Eupolyphaga Seu Steleophaga* (Tubiechong),	Regulating the menstrual function	Qian-jin-yao-fang

	Hirudo (Shuizhi), *Zingiberis Rhizoma* (Ganjiang), *Moutan cortex* (Mudanpi), *Aconiti lateralis radix praeparata* (Fuzi), *Gleditsiae abnormalis fructus* (Zaojia), *Angelicae sinensis radix* (Danggui), *Paeoniae radix alba* (Baishao), *Glycyrrhizae radix et rhizoma* (Gancao), *Oreoselinum* (Fangkui), *Holotrichiadiomphalia Bates* (Qicao), *Tabanus* (Mengchong), *Rhei radix et rhizoma* (Dahuang)		
Dahuang Zhechong Wan	*Rhei radix et rhizoma* (Dahuang), *Scutellariae radix* (Huangqin), *Glycyrrhizae radix et rhizoma* (Gancao), *Persicae semen* (Taoren), *Armeniacae semen amarum* (Kuxingren), *Paeoniae radix alba* (Baishao), *Rehmanniae radix* (Dihuang), *Toxicodendri resina* (Ganqi), *Tabanus* (Mengchong), ***Hirudo*** (Shuizhi), *Holotrichiadiomphalia Bates* (Qicao), *Eupolyphaga seu seleophaga* (Tubiechong).	Promoting blood circulation and removing stasis	Zhang-si-yi-tong
Dahuang Wan	*Rhei radix et rhizome* (Dahuang), *Persicae semen* (Taoren), *Toxicodendri resina* (Ganqi), *Smilacis glabrae rhizoma* (Tufuling), *Letidii semen* (Tinglizi), *Achyranthis bidentatae radix* (Niuxi), *Moutan cortex* (Mudanpi), *Hirudo* (Shuizhi), *Chuanxiong Rhizoma* (Chuanxiong), *Cinnamomi cortex* (Rougui), *Bupleuri radix* (Chaihu), *Rubia yunnanensis Diels* (Zishen), *Ginseng radix et rhizoma* (Renshen), *Angelicae sinensis radix* (Danggui), *Zingiberis rhizoma* (Ganjiang), *Tabanus* (Mengchong), *Zanthoxylum Bungeanum Pericarpium* (Huajiao), *Eupolyphaga seu steleophaga* (Tubiechong), *Euodiae fructus* (Wuzhuyu), *Rehmanniae radix* (Dihuang)	Regulating the menstrual function	Tai-ping-sheng-hui-Fang
Damengchong Wan	*Tabanus* (Mengchong), *Holotrichiadiomphalia bates* (Qicao), *Rehmanniae radix* (Dihuang), *Moutan cortex* (Mudanpi), *Toxicodendri resina* (Ganqi), *Paeoniae radix alba* (Baishao), *Achyranthis bidentatae radix* (Niuxi), *Trichosanthes Cucumeroides Radix* (Tuguagen), *Cinnamomi cortex* (Rougui), *Euodiae fructus* (Wuzhuyu), *Persicae semen* (Taoren), *Scutellariae radix* (Huangqin), *Rubia yunnanensis* Diels (Zishen), *Poria* (Fuling), *Sargassum* (Haizao), ***Hirudo*** (Shuizhi), *Natrii Sulfas* (Mangxiao), *Ginseng radix et rhizoma* (Renshen),	Regulating the menstrual function	Qian-jin-yao-fang

	Letidii semen (Tinglizi).		
Danggui Wan	*Angelicae sinensis radix* (Danggui), *Letidii semen* (Tinglizi), *Aconiti lateralis radix praeparata* (Fuzi), *Euodiae fructus* (Wuzhuyu), *Rhei radix et rhizoma* (Dahuang), *Scutellariae radix* (Huangqin), *Cinnamomi cortex* (Rougui), *Zingiberis rhizoma* (Ganjiang), *Moutan cortex* (Mudanpi), *Chuanxiong rhizoma* (Chuanxiong), *Asari radix et rhizoma* (Xinxi), *Zanthoxylum bungeanum pericarpium* (Huajiao), *Bupleuri radix* (Chaihu), *Magnoliae officinalis cortex* (Houpo), *Rubia yunnanensis* Diels (Zishen), *Glycyrrhizae radix et rhizoma* (Gancao), *Tabanus* (Mengchong), *Hirudo* (Shuizhi)	Promoting blood circulation and removing stasis	Qian-jin-yao-fang
Didang Wan	*Hirudo* (Shuizhi), *Tabanus* (Mengchong), *Persicae semen* (Taoren), *Rhei radix et rhizoma* (Dahuang)	Promoting blood circulation and removing stasis	Dan-xi-xin-fa
Dihuang Tongjing Wan	*Rehmanniae radix praeparata* (shudihuang) ,*Tabanus* (Mengchong), *Hirudo* (SHuizhi), *Persicae semen* (Taoren)	Promoting blood circulation and removing stasis	Yi-xue-zheng-zhuan
Dingtong Wan	*Clematidis radix et rhizoma* (Weilingxian), *Momordicae semen* (Mubiezi), *Aconiti radix* (Chuanwu), *Saposhnikoviae radix* (Fangfeng), *Angelicae dahuricae radix* (Baizhi), *Faeces trogopterori* (Wulingzhi), *Pheretima* (Dilong), *Hirudo* (Shuizhi), *Cinnabaris* (Zhusha)	Promoting blood circulation and removing stasis	Feng-ke-ji-yan-ming-fan g
Fangkui Wan	*Oreoselinum* (Fangkui), *Myrrha* (Moyao), *Toxicodendri resina* (Ganqi), *Halite violaceous* (Naosha), *Hirudo* (Shuizhi), *Dogbile* (Goudan), *Curcumae longae rhizome* (Jianghuang), *Genkwa flos* (Yuanhua)	Promoting blood circulation and removing stasis, stimulating menstruation discharge	Tai-ping-sheng-hui-Fang
Gandihuang Danggui Wan	*Rehmanniae radix* (Dihuang), *Angelicae sinensis radix* (Danggui), *Glycyrrhizae radix et rhizoma* (Gancao), *Achyranthis bidentatae radix* (Niuxi), *Paeoniae radix alba* (Baishao), *Zingiberis rhizoma* (Ganjiang), *Lycopi herba* (Zelan), *Ginseng radix et rhizoma* (Renshen), *Moutan cortex* (Mudanpi), *Salviae miltiorrhizae radix et rhizoma* (Danshen), *Zanthoxylum bungeanum pericarpium* (Huajiao), *Angelicae dahuricae radix* (Baizhi), *Scutellariae radix*	Promoting blood circulation and removing stasis, stimulating menstruation discharge	Qian-jin-yao-fang

	(Huangqin), *Cinnamomi cortex* (Rougui), *Eupolyphaga seu steleophaga* (Tubiechong), *Chuanxiong rhizoma* (Chuanxiong), *Persicae semen* (Taoren), *Hirudo* (Shuizhi), *Tabanus* (Mengchong), *Typhae pollen* (Puhuang)		
Ganjiang Wan	*Zingiberis rhizoma* (Ganjiang), *Bupleuri radix* (Chaihua), *Paeoniae radix rubra* (Chishao), *Ginseng radix et rhizoma* (Renshen), *Zanthoxylum bungeanum pericarpium* (Huajiao), *Natrii sulfas* (Xiaoshi), *Rhei radix et rhizoma* (Dahuang), *Angelicae sinensis radix* (Danggui), *Armeniacae semen amarum* (Kuxingren), *Chuanxiong rhizoma* (Chuanxiong), *Hirudo* (Shuizhi), *Tabanus* (Mengchong), *Persicae semen* (Taoren), *Smilacis glabrae rhizoma* (Tufuling), *Holotrichiadiomphalia bates* (Qicao), *Eupolyphaga seu steleophaga* (Tubiechong)	Promoting blood circulation and removing stasis, stimulating menstruation discharge	Tai-ping-sheng-hui-Fang
Ganqi Wan	*Toxicodendri resina* (Ganqi), *Moutan cortex* (Mudanpi), *Belamcandae rhizoma* (Shegan), *Scutellariae radix* (Huangqin), *Persicae semen* (Taoren), *Cinnamomi cortex* (Rougui), *Euodiae fructus* (Wuzhuyu), *Rhei radix et rhizoma* (Dahuang), *Hirudo* (Shuizhi), *Bupleuri radix* (Chaihua), *Artemisia keiskeana fructus* (Anzi), *Tabanus* (Mengchong), *Crinis carbonisatus* (Xueyutan), *Eupolyphaga seu steleophaga* (Tubiechong), *Holotrichiadiomphalia bates* (Qicao), *Cannabis fructus* (Huomaren), *Trionycis carapax* (Biejia)	Regulating the menstrual function. Treating female infertility	Tai-ping-sheng-hui-Fang
Goudan Wan	*Dogbile* (Goudan), *Halite violaceous* (Naosha), *Toxicodendri resina* (Ganqi), *Genkwa flos* (Yuanhua), *Corydalis yanhusuo* (Yanhusuo), *Zingiberis rhizoma* (Ganjiang), *Mylabris* (Banmao), *Angelicae sinensis radix* (Danggui), *Draconis sanguis* (Xuejie), *Arsenicum sublimatum* (Pishuang), *Terra flava usta* (Fulonggan), *Pyritum* (Zirantong), *Tabanus* (Menghocng), *Hirudo* (Shuizhi)	Promoting blood circulation and removing stasis, stimulating menstruation discharge	Tai-ping-sheng-hui-Fang
Guijian Wan	*Euonymus alatu ramulus* (Guijianyu), *Natrii sulfas* (Mangxiao), *Bupleuri radix* (Chaihua), *Hirudo* (Shuizhi), *Tabanus* (Mengchong), *Rhei radix et rhizoma* (Dahuang), *Smilacis glabrae*	Promoting blood circulation and removing stasis, stimulating	Tai-ping-sheng-hui-Fang

	rhizoma (Tufuling), *Toxicodendri resina* (Ganqi), *Zanthoxylum bungeanum pericarpium* (Huajia), *Letidii semen* (Tinglizi), *Armeniacae semen amarum* (Kuxingren), *Persicae semen* (Taoren), *Moutan cortex* (Mudanpi)	menstruation discharge	
Haige Wan	*Concha meretricis seu cyclinae* (Geke), *Angelicae sinensis radix* (Danggui), *Lygodii spora* (Haijinsha), *Calomelas* (Qingfen), *Sargassum* (Haizao), ***Hirudo*** (Shuizhi), *Indigo naturalis* (Qingdai), *Talcum* (Huashi), *Olibanum* (Ruxiang), *Cinnabaris* (Zhusha), *Elephantopus scaber* (Didan)	Promoting blood and Qi circulation	Ji-sheng-ba-cui
Heisheng Wan	*Crinis carbonisatus* (Xueyutan), *Carp skin carbonisatus* (Chiliyupi), *Tabanus* (Mengchong), ***Hirudo*** (Shuizhi), *Sojae semen nigrum* (Heidou), *Saigae tataricae cornu* (Lingyangjiao), *Draconis sanguis* (Xuejie), *Crotonis fructus* (Badou)	Promoting blood circulation and eliminating blood stasis	Tai-ping-sheng-hui-Fang
Hupojian Wan	*Succinum* (Hupo), *Tabanus* (Mengchong), ***Hirudo*** (Shuizhi), *Cinnamomi cortex* (Rougui), *Persicae semen* (Taoren), *Rhei radix et rhizoma* (Dahuang)	stimulating menstruation discharge	Tai-ping-sheng-hui-Fang
Hupo Wan	*Succinum* (Hupo), *Myrrha* (Moyao), *Angelicae sinensis radix* (Danggui), *Paeoniae radix rubra* (Chishao), *Sparganii rhizoma* (Sanleng), *Trionycis carapax* (Biejia), *Tabanus* (Mengchong), ***Hirudo*** (Shuizhi)	Promoting blood circulation and removing stasis, stimulating menstruation discharge	Tai-ping-sheng-hui-Fang
Huashi Wan	*Akebiae caulis* (Mutong), *Talcum* (Huashi), *Dianthi herba* (Qumai), *Lygodii spora* (Haijingsha), *Kansui radix* (Gansui), *Tetrapanacis medulla* (Tongcao), ***Hirudo*** (Shuizhi), *Elephantopus scaber* (Didan)	Promoting Qi circulation, clearing damp	Ji-feng-pu-ji-fang
Huagu Wan	*Sparganii rhizoma* (Sanleng), *Curcumae rhizoma* (Ezhu), *Toxicodendri resina* (Ganqi), *Halite violaceous* (Naosha), *Tabanus* (Mengchong), ***Hirudo*** (Shuizhi), *Succinum* (Hupo), *Achyranthis bidentatae radix* (Niuxi), *Cinnamomi cortex* (Rougui), *Rhei radix et rhizoma* (Dahuang)	Promoting blood circulation and removing stasis	Shou-shi-bao-yuan

Jianxian Wan<br><br>Jianxian San	*Aconiti lateralis radix praeparata* (Fuzi), *Euonymus alatus ramulus* (Guijianyu), *Fluoritum* (Zishiying), *Alismatis rhizoma* (Zexie), *Cinnamomi cortex* (Rougui), *Corydalis yanhusuo* (Yanhusuo), *Aucklandiae radix* (Muxiang), *Arecae semen* (Binlang), *Draconis sanguis* (Xuejie), *Rhei radix et rhizoma* (Dahuang), *Persicae semen* (Taoren), *Sparganii rhizoma* (Sanleng), ***Hirudo*** (Shuizhi)	Promoting blood circulation and removing stasis	Lei-zheng-zhi-cai<br><br>Fu-ke-yu-chi
Jianghuang Wan	*Curcumae longae rhizoma* (Jianghuang), *Moutan cortex* (Mudanpi), *Paeoniae radix rubra* (Chishao), *Cinnamomi cortex* (Rougui), *Genkwa flos* (Yuanhua), *Angelicae sinensis radix* (Danggui), *Trionycis carapax* (Biejia), *Succinum* (Hupo), *Corydalis yanhusuo* (Yanhusuo), *Euonymus alatus ramulus* (Guijianyu), *Aucklandiae radix* (Muxiang), *Halite violaceous* (Naosha), *Campsis flos* (Lingxiaohua), *Sparganii rhizoma* (Sanleng), ***Hirudo*** (Shuizhi), *Tabanus* (Mengchong), *Rhei radix et rhizoma* (Dahuang), *Toxicodendri resina* (Ganqi)	Promoting Qi circulation and stimulating menstruation discharge	Tai-ping-sheng-hui-Fang
Lingxiaohua Wan	*Campsis flos* (Lingxiaohua), *Myrrha* (Moyao), *Persicae semen* (Taoren), ***Hirudo*** (Shuizhi), *Talcum* (Huashi), *Halite violaceous* (Naosha), *Mylabris* (Banmao), *Dogbile* (Goudan)	Promoting blood circulation and stimulating menstruation discharge	Tai-ping-sheng-hui-Fang
Linglijia Wan	*Scales carps* (Linglijia), *Agkistrodon* (Fushe), *Mylabris* (Banmao), *Scolopendra* (Wugong), *Concha meretricis seu cyclinae* (Geke), *Tabanus* (Mengchong), ***Hirudo*** (Shuizhi), *Spider* (Zhizhu), *Holotrichiadiomphalia bates* (Qicao), *Aconiti lateralis radix praeparata* (Fuzi), *Omphalia* (Leiwan), *Mercury* (Shuiyin), *Natrii sulfas* (Mangxiao), *Rhei radix et rhizoma* (Dahuang), *Gypsum fibrosum* (Shigao), *Crotonis fructus* (Badou), *Cinnamomi cortex* (Rougui), *Zanthoxylum bungeanum pericarpium* (Huajiao), *Natrii sulfas* (Mangxiao), *Dens draconis* (Longgu), *Alumen* (Fanshihui), *Coptidis rhizoma* (Huanglian), *Talcum* (Huashi)	Treating gafeira	Tai-ping-sheng-hui-Fang
Moyao Wan	*Angelicae sinensis radix* (Danggui), *Paeoniae radix Alba* (Chishao), *Cinnamomi cortex* (Rougui), *Persicae semen* (Taoren), *Myrrha* (Moyao), *Tabanus* (Mengchong), ***Hirudo***	Promoting blood circulation and removing stasis, promoting Qi to	Fu-ke-yu-chi

	(Shuizhi)	relieve pain	
Mengchong Wan	*Tabanus* (Mengchong), *Persicae semen* (Taoren), *Mantidis ootheca* (Sangpiaoxiao), *Holotrichiadiomphalia bates* (Qicao), *Haematitum* (Daizheshi), ***Hirudo*** (Shuizhi), *Rhei radix et rhizoma* (Dahuang)	Promoting blood circulation and stimulating menstruation discharge	Tai-ping-sheng-hui-Fang
Mudan Wan	*Moutan cortex* (Mudanpi), *Rhei radix et rhizoma* (Dahuang), *Paeoniae radix rubra* (Chishao), *Aucklandiae radix* (Muxiang), *Persicae semen* (Taoren), *Tabanus* (Mengchong), ***Hirudo*** (Shuizhi), *Holotrichiadiomphalia bates* (Qicao), *Dianthi herba* (Qumai), *Chuanxiong rhizoma* (Chuanxiong), *Angelicae sinensis radix* (Danggui), *Sargassum* (Haizao), *Cinnamomi cortex* (Rougui)	Promoting blood circulation and stimulating menstruation discharge	Tai-ping-sheng-hui-Fang
Muli Wan	*Ostreae concha* (Muli), *Rhei radix et rhizoma* (Dahuang), *Bupleuri radix* (Chaihu), *Natrii sulfas* (Mangxiao), *Zingiberis rhizoma* (Ganjiang), *Chuanxiong rhizoma* (Chuanxiong), *Zanthoxylum bungeanum pericarpium* (Huajiao), *Smilacis glabrae rhizoma* (Tufuling), *Letidii semen* (Tinglizi), ***Hirudo*** (Shuizhi), *Armeniacae semen amarum* (Kuxingren), *Tabanus* (Mengchong), *Persicae semen* (Taoren)	Promoting blood circulation and stimulating menstruation discharge	Tai-ping-sheng-hui-Fang
Muxiang Wan	*Aucklandiae radix* (Muxiang), *Sparganii rhizoma* (Sanleng), *Arecae semen* (Binlang), *Cinnamomi cortex* (Rougui), *Aconiti lateralis radix praeparata* (Fuzi), *Myrrha* (Moyao), *Ferulae resina* (Awei), *Persicae semen* (Taoren), *Trionycis carapax* (Biejia), *Chuanxiong rhizoma* (Chuanxiong), *Tabanus* (Mengchong), ***Hirudo*** (Shuizhi), *Angelicae sinensis radix* (Danggui), *Moutan cortex* (Mudanpi), *Paeoniae radix rubra* (Chishao), *Halite violaceous* (Naosha), *Rhei radix et rhizoma* (Dahuang), *Toxicodendri resina* (Ganqi)	Treating postpartum blood stasis	Tai-ping-sheng-hui-Fang
Naoshajian Wan	*Halite violaceous* (Naosha), *Toxicodendri resina* (Ganqi), *Rhei radix et rhizoma* (Dahuang), *Trionycis carapax* (Biejia), *Myrrha* (Moyao), *Faeces trogopterori* (Wulingzhi), *Dogbile* (Goudan), *Mylabris* (Banmao), ***Hirudo*** (Shuizhi), *Crotonis fructus* (Badou)	Promoting blood circulation and removing stasis, promoting Qi to relieve pain	Tai-ping-sheng-hui-Fang

Naosha Wan	*Halite violaceous* (Naosha), *Sulfur* (Liuhuang), *Genkwa flos* (Yuanhua), *Myrrha* (Moyao), ***Hirudo*** (Shuizhi), *Angelicae sinensis radix* (Dahuang), *Rhei radix et rhizoma* (Dahuang), *Moutan cortex* (Mudanpi), *Tabanus* (Mengchong)	Promoting blood circulation and removing stasis	Tai-ping-sheng-hui-Fang
Niuxi Wan	*Achyranthis bidentatae radix* (Niuxi), *Angelicae sinensis radix* (Danggui), *Atractylodis macrocephalae rhizoma* (Baizhu), *Chuanxiong rhizoma* (Chuanxiong), *Cinnamomi cortex* (Rougui), *Persicae semen* (Taoren), *Rhei radix et rhizoma* (Dahuang), ***Hirudo*** (Shuizhi), *Euonymus alatus ramulus* (Guijianyu)	Promoting blood circulation and stimulating menstruation discharge	Tai-ping-sheng-hui-Fang
Pengsha Wan	*Borax* (Pengsha), *Chloriti lapis* (Qingmengshi), *Squama manitis* (Chuanshanjia), *Magnetitum* (Cishi), *Sparganii rhizoma* (Sanleng), *Toxicodendri resina* (Ganqi), *Tabanus* (Mengchong), ***Hirudo*** (Shuizhi), *Crotonis fructus* (Badou), *Halloysitum rubrum* (Chishizhi)	Promoting blood and Qi circulation	Tai-ping-sheng-hui-Fang
Poyu Dan	***Hirudo*** (Shuizhi), *Angelicae sinensis radix* (Danggui), *Paeoniae radix alba* (Baishao), *Poria* (Fuling), *Cinnamomi cortex* (Rougui), *Persicae semen* (Taoren), *Rehmanniae radix* (Dihuang), *Aurantii fructus* (Zhiqiao), *Polypopus* (Zhuling)	Eliminating blood stasis	Shang-han-bian-zheng-l u
Qi'ao Wan	*Rhei radix et rhizoma* (Dahuang), *Peucedani radix* (Qianhu), *Natrii sulfas* (Mangxiao), *Zingiberis rhizoma* (Ganjiang), *Poria* (Fuling), *Armeniacae semen amarum* (Kuxingren), *Zanthoxylum bungeanum pericarpium* (Huajiao), *Letidii semen* (Tinglizi), *Persicae semen* (Taoren), ***Hirudo*** (Shuizhi), *Tabanus* (Mengchong)	Stimulating menstruation discharge relieving uneasiness of mind and body tranquilization	Qian-jin-yi-fang
Qicao Wan	*Holotrichiadiomphalia bates* (Qicao), *Tabanus* (Mengchong), ***Hirudo*** (Shuizhi), *Mantidis ootheca* (Sangpiaoxiao), *Dogbile* (Goudan), *Haematitum* (Daizheshi), *Rhei radix et rhizoma* (Dahuang), *Persicae semen* (Taoren)	Stimulating menstruation discharge	Tai-ping-sheng-hui-Fang
Qilinjie Wan	*Draconis sanguis* (Xuejie), *Myrrha* (Moyao), *Halite violaceous* (Naosha), *Dogbile* (Goudan), *Toxicodendri resina* (Ganqi), *Genkwa flos* (Yuanhua), *Corydalis yanhusuo* (Yanhusuo), *Aconiti lateralis radix praeparata* (Fuzi), *Aconiti radix* (Chuanwu), *Angelicae sinensis radix*	Promoting blood circulation and removing stasis, promoting Qi to	Tai-ping-sheng-hui-Fang

	(Danggui), *Arsenicum sublimatum* (Pishuang), *Terra flava usta* (Fulonggan), *Tabanus* (Mengchong), ***Hirudo*** (Shuizhi), *Crotonis fructus* (Badou)	relieve pain	
Qinjiao Wan	*Gentianae macrophylilae radix* (Qinjiao), *Scrophulariae radix* (Xuanshen), *Ginseng radix et rhizoma* (Renshen), *Ampelopsis radix* (Bailian), *Armadillidium vulgare* (Shufu), *Angelicae dahuricae radix* (Baizhi), *Astragali radix* (Huangqi), *Platycodonis radix* (Jiegeng), *Vespae nidus* (Fengfang), *Bombyx batryticatus* (Jiangchan), *Persicae semen* (Taoren), *Holotrichiadiomphalia bates* (Qicao), *Cynanchi atrati radix et rhizoma* (Baiwei), *Asari radix et rhizoma* (Xixin), *Ulmus macrocarpae fructus* (Wuyi), *Rubia yunnanensis* Diels (Zishen), *Adenophorae radix* (Nanshashen), *Saposhnikoviae radix* (Fangfeng), *Glycyrrhizae radix et rhizoma* (Gancao), *Moutan cortex* (Mudanpi), *Achyranthis bidentatae radix* (Niuxi), *Selaginellae herba* (Juanbai), *Schisandrae chinensis fructus* (Wuweizi), *Paeoniae radix alba* (Baishao), *Cinnamomi cortex* (Rougui), *Rhei radix et rhizoma* (Dahuang), *Dendrobii caulis* (Shihu), *Atractylodis macrocephalae rhizoma* (Baizhu), *Platycladi semen* (Baiziren), *Poria* (Fuling), *Angelicae sinensis radix* (Danggui), *Zingiberis rhizoma* (Ganjiang), *Lycopi herba* (Zelan), *Rehmanniae radix* (Dihuang), *Chuanxiong rhizoma* (Chuanxiong), *Toxicodendri resina* (Ganqi), *Whitequartz* (Baishiying), *Fluoritum* (Zishiying), *Aconiti lateralis radix praeparata* (Fuzi), *Stalactitum* (Zhongrushi), ***Hirudo*** (Shuizhi), *Tabanus* (Mengchong), *Tallmonkshoodroot* (Mabu)	Regulating menstrual function. Treating female infertility	Qian-jin-yao-fang
Shenxiao Lingxiaohua Wan	*Campsis flos* (Lingxiaohua), *Genkwa flos* (Yuanhua), *Sparganii rhizoma* (Sanleng), *Aucklandiae radix* (Muxiang), *Curcumae longae rhizoma* (Jianghuang), ***Hirudo*** (Shuizhi), *Halite violaceous* (Naosha), *Mylabris* (Banmao)	Promoting blood circulation and removing stasis, regulating menstrual function	Tai-ping-sheng-hui-Fang
Shenggandihuang Wan	*Rehmanniae radix* (Dihuang), *Persicae semen* (Taoren), *Angelicae sinensis radix* (Danggui), *Achyranthis bidentatae radix* (Niuxi), *Rhei radix et rhizoma* (Dahuang), *Chuanxiong rhizoma* (Chuanxiong), *Trichosanthes cucumeroides radix* (Tuguagen), *Paeoniae radix rubra* (Chishao), *Cinnamomi cortex* (Rougui), *Natrii sulfas* (Mangxiao), *Tabanus* (Mengchong),	Regulating the menstrual function and relieving pain	Tai-ping-sheng-hui-Fang

	Hirudo (Shuizhi)		
Shengjin Wan	*Mercury* (Shuiyin)*, Sulfur* (Liuhuang)*, Trachycarpus wagnerianus cortex* (Zonglvpi)*, Toxicodendri resina* (Ganqi)*, Scales carps* (Liyulin)*, Pyritum* (Zirantong)*, Dogbile* (Goudan)*, Draconis sanguis* (Xuejie)*, Angelicae sinensis radix* (Danggui)*, Corydalis yanhusuo* (Yanhusuo)*, **Hirudo*** (Shuizhi)*, Tabanus* (Mengchong)*, Zaocys* (Wushaoshe)*, Cinnamomi cortex* (Rougui)*, Crinis carbonisatus* (Xueyutan)*, Myrrha* (Moyao)	Eliminating blood stasis to relieve pain	Tai-ping-sheng-hui-Fang
Taohua Wan	*Prunus persica Flos* (Taohua)*, Styrax* (Suhexiang)*, Benzoinum* (Anxixiang)*, Aucklandiae radix* (Muxiang)*, Arecae semen* (Binlang)*, Natrii sulfas* (Mangxiao)*, **Hirudo*** (Shuizhi)*, Tabanus* (Mengchong)*, Trionycis carapax* (Biejia)*, Draconis sanguis* (Xuejie)*, Aconiti lateralis radix praeparata* (Fuzi)*, Bupleuri radix* (Chaihu)*, Selaginellae herba* (Juanbai)*, Angelicae sinensis radix* (Danggui)*, magnolia flos* (Xinyi)*, Angelicae dahuricae radix* (Baizhi)*, Fluoritum* (Zishiying)*, Limonitum* (Yuyuliang)*, Chuanxiong rhizoma* (Chuanxiong)*, Moutan cortex* (Mudanpi)*, Asari radix et rhizoma* (Xixin)*, Ophiopogonis radix* (Maidong)*, Notopterygii rhizoma et radix* (Qianghuo)*, Cinnamomi cortex* (Rougui)*, Myristicae semen* (Roudoukou)	Regulating menstrual function, Treating female infertility	Tai-ping-sheng-hui-Fang
TuguagenWan	*Holotrichiadiomphalia bates* (Qicao)*, Rehmanniae radix praeparata* (Shudihuang)*, Moutan cortex* (Mudanpi)*, Toxicodendri Resina* (Ganqi)*, Paeoniae radix rubra* (Chishao)*, Achyranthis bidentatae radix* (Niuxi)*, Trichosanthes cucumeroides radix* (Tuguagen)*, Cinnamomi cortex* (Rougui)*, Persicae semen* (Taoren)*, Scutellariae radix* (Huangqin)*, Rubia yunnanensis* Diels (Zishen)*, Sargassum* (Haizao)*, Poria* (Fuling)*, Tabanus* (Mengchong)*, **Hirudo*** (Shuizhi)*, Natrii sulfas* (Mangxiao)*, Ginseng radix et rhizoma* (Renshen)*, Euodiae fructus* (Wuzhuyu)	Promoting blood and Qi circulation for pain relief	Ji-feng-pu-ji-fang
Weipi Wan	*Corium erinacei* (Weipi)*, Concha meretricis seu cyclinae* (Geke)*, Tabanus* (Mengchong)*, Holotrichiadiomphalia bates* (Qicao)*, Scales carps* (Linglijia)*, Mylabris* (Banmao)*, Scolopendra* (Wugong)*, Aconiti lateralis radix praeparata* (Fuzi)*, Spider* (Zhizhu)*, **Hirudo*** (Shuizhi)*, Crotonis fructus* (Badou)*, Omphalia* (Leiwan)*, Mercury* (Shuiyin)*, Rhei radix et*	Treating gafeira	Tai-ping-sheng-hui-Fang

	rhizoma (Dahuang), *Cinnabaris* (Zhusha), *Cinnamomi cortex* (Rougui), *Coptidis rhizoma* (Huanglian), *Gypsum fibrosum* (Shigao), *Natrii sulfas* (Mangxiao), *Dens draconis* (Longgu), *Zanthoxylum bungeanum pericarpium* (Huajia), *Kansui radix* (Gansui), *Alumen* (Baifanhui), *Talcum* (Huashi)		
Wulai Wan	*Corium erinacei* (Weipi), *Concha meretricis seu cyclinae* (Geke), *Agkistrodon* (Fushe), *Tabanus* (Mengchong), *Holotrichiadiomphalia bates* (Qicao), *Scales carps* (Linglijia), *Mylabris* (Banmao), *Scolopendra* (Wugong), *Aconiti lateralis radix praeparata* (Fuzi), *Spider* (Zhizhu), ***Hirudo*** (Shuizhi), *Omphalia* (Leiwan), *Crotonis fructus* (Badou), *Mercury* (Shuiyin), *Rhei radix et rhizoma* (Dahuang), *Cinnamomi cortex* (Rougui), *Coptidis rhizoma* (Huanglian), *Gypsum fibrosum* (Shigao), *Zanthoxylum bungeanum pericarpium* (Huajiao), *Natrii sulfas* (Mangxiao), *Dens draconis* (Longgu), *Kansui radix* (Gansui), *Talcum* (Huashi)		Wai-tai-mi-yao
Xijiao Wan	*Cornu rhinocerotis* (Xijiao), ***Hirudo*** (Shuizhi), *Glycyrrhizae radix et rhizoma* (Gancao), *Sojae semen nigrum* (Heidou)	Treating anal fistula	Tai-ping-sheng-hui-Fang
Xiaoshida Wan	*Natrii sulfas* (Mangxiao), *Zanthoxylum bungeanum pericarpium* (Huajiao), ***Hirudo*** (Shuizhi), *Tabanus* (Mengchong), *Rhei radix et rhizoma* (Dahuang), *Poria* (Fuling), *Bupleuri radix* (Chaihu), *Chuanxiong rhizoma* (Chuanxiong), *Holotrichiadiomphalia bates* (Qicao)	Promoting blood circulation and removing stasis, clearing heat and detoxifying	Qian-jin-yi-fang
Xuegu Wan	*Sparganii rhizoma* (Sanleng), *Curcumae rhizoma* (Ezhu), *Toxicodendri resina* (Ganqi), *Achyranthis bidentatae radix* (Niuxi), *Tabanus* (Mengchong), ***Hirudo*** (Shuizhi), *Cinnamomi cortex* (Rougui), *Succinum* (Hupo), *Rhei radix et rhizoma* (Dahuang), *Halite violaceous* (Naosha)	Promoting blood circulation and removing stasis	Shang-bian-qi-fang
Yuanhuajian Wan	*Genkwa flos* (Yuanhua), *Halite violaceous* (Naosha), *Achyranthis bidentatae radix* (Niuxi), *Angelicae sinensis radix* (Danggui), *Paeoniae radix rubra* (Chishao), *Citri reticulatae pericarpium* (Qingpi), *Tabanus* (Mengchong), *Aucklandiae radix* (Muxiang), ***Hirudo***	Promoting blood circulation, removing stasis, stimulating menstruation discharge for pain	Tai-ping-sheng-hui-Fang

	(Shuizhi), *Rhei radix et rhizoma* (Dahuang), *Cinnamomi cortex* (Rougui), *Succinum* (Hupo)	relief	
Zaizao Wan	*Hawksbill* (Daimao), *Borneolum syntheticum* (Bingpian), ***Hirudo*** (Shuizhi), *Scolopendra* (Wugong), *Ephedrae herba* (Mahuang)	Clearing heat and detoxifying	Yi-fang-kao
Buqi Xierong Tang	*Cimicifugae rhizoma* (Shengma), *Forsythiae fructus* (Lianqiao), *Rehmanniae radix* (Dihuang), *Scutellariae radix* (Huangqin), *Angelicae sinensis radix* (Danggui), *Sappan lignum* (Sumu), *Scorpio* (Quanxie), *Pheretima* (Dilong), *Astragali Radix* (Huangqi), *Coptidis rhizoma* (Huanglian), *Platycodonis radix* (Jiegeng), *Glycyrrhizae radix et rhizoma* (Gancao), *Ginseng radix et rhizoma* (Renshen), *Populi resina* (Hutonglei), *Persicae semen* (Taoren), *Moschus* (Shexiang), *Tabanus* (Mengchong), ***Hirudo*** (Shuizhi)	Activating qi for relieving qi stagnation, clearing heat and detoxifying	Yi-fang-kao
Chaihu Cong'er Tang	*Forsythiae fructus* (Lianqiao), *Bupleuri radix* (Chaihu), *Glycyrrhizae radix et rhizoma* (Gancao), *Angelicae sinensis radix* (Danggui), *Ginseng radix et rhizome* (Renshen), ***Hirudo*** (Shuizhi), *Tabanus* (Mengchong), *Moschus* (Shexiang)		Yi-xue-zheng-zhuan
Dahuang PuxiaoTang	*Rhei radix et rhizoma* (Dahuang), *Achyranthis bidentatae radix* (Niuxi), *Natrii sulfas* (Mangxiao), *Moutan cortex* (Mudanpi), *Glycyrrhizae radix et rhizoma* (Gancao), *Asteris radix et rhizoma* (Ziwan), *Haematitum* (Daizheshi), *Persicae semen* (Taoren), *Tabanus* (Mengchong), ***Hirudo*** (Shuizhi), *Zingiberis rhizoma* (Ganjiang), *Asari radix et rhizoma* (Xixin), *Natrii sulfas* (Mangxiao), *Cannabis fructus* (Huomaren)	Regulating menstrual function	Qian-jin-yao-fang
Dangpao Tang	*Natrii sulfas* (Mangxiao), *Persicae semen* (Taoren), *Poria* (Fuling), *Moutan cortex* (Mudanpi), *Rhei radix et rhizoma* (Dahuang), *Ginseng radix et rhizoma* (Renshen), *Cinnamomi cortex* (Rougui), *Paeoniae radix alba* (Baishao), *Magnoliae officinalis cortex* (Houpo), *Asari radix et rhizoma* (Xixin), *Achyranthis bidentatae radix* (Niuxi), *Angelicae sinensis radix* (Danggui), *Citri reticulatae pericarpium* (Chenpi), *Aconiti lateralis radix praeparata* (Fuzi), *Tabanus* (Mengchong), ***Hirudo*** (Shuizhi)	Treating female infertility	Qian-jin-yi-fang

Huangqin Mudna Tang	*Scutellariae radix* (Huangqin), *Moutan cortex* (Mudanpi), *Persicae semen* (Taoren), *Dianthi herba* (Qumai), *Chuanxiong rhizoma* (Chuanxiong), *Paeoniae radix alba* (Baishao), *Aurantii fructus immaturus* (Zhishi), *Belamcandae rhizoma* (Shegan), *Sargassum* (Haizao), *Rhei radix et rhizoma* (Dahuang), *Tabanus* (Mengchong), ***Hirudo*** (Shuizhi), *Holotrichiadiomphalia bates* (Qicao)	Promoting blood circulation, stimulating menstruation discharge	Qian-jin-yao-fang
Mangxiao Tang	*Natrii sulfas* (Mangxiao), *Cinnabaris* (Zhusha), *Angelicae sinensis radix* (Danggui), *Paeoniae radix alba* (Baishao), *Trichosanthes cucumeroides radix* (Tuguagen), ***Hirudo*** (Shuizhi), *Rhei radix et rhizoma* (Dahuang), *Persicae semen* (Taoren)	Stimulating menstruation discharge	Qian-jin-yao-fang
Mudan Dahuang tang	*Moutan cortex* (Mudanpi), *Rhei radix et rhizoma* (Dahuang), *Natrii sulfas* (Mangxiao), *Persicae semen* (Taoren), *Ginseng radix et rhizoma* (Renshen), *Poria* (Fuling), ***Hirudo*** (Shuizhi), *Tabanus* (Mengchong), *Glycyrrhizae radix et rhizoma* (Gancao)	Regulating menstrual function for pain relief	Qian-jin-yi-fang
Poxue Santeng Tang	*Notopterygii rhizoma et radix* (Qianghuo), *Saposhnikoviae radix* (Fangfeng), *Cinnamomi cortex* (Rougui), *Sappan lignum* (Sumu), *Forsythiae fructus* (Lianqiao), *Angelicae sinensis radix* (Danggui), *Bupleuri radix* (Chaihu), ***Hirudo*** (Shuizhi), *Moschus* (Shexiang)	Treating traumatic injury and blood stasis	Lan-shi-mi-cang
Puxiao Dangpao Tang	*Natrii sulfas* (Mangxiao), *Moutan cortex* (Mudanpi), *Angelicae sinensis radix* (Danggui), *Rhei radix et rhizoma* (Dahuang), *Persicae semen* (Taoren), *Asari radix et rhizoma* (Xixin), *Magnoliae officinalis cortex* (Houpo), *Platycodonis radix* (Jiegeng), *Paeoniae radix rubra* (Chishao), *Ginseng radix et rhizoma* (Renshen), *Poria* (Fuling), *Cinnamomi cortex* (Rougui), *Glycyrrhizae radix et rhizoma* (Gancao), *Achyranthis bidentatae radix* (Niuxi), *Citri reticulatae pericarpium* (Chenpi), *Tabanus* (Mengchong), ***Hirudo*** (Shuizhi), *Aconiti lateralis radix praeparata* (Fuzi)	Treating female infertility	You-you-xin-shu
Sanyu Tang	***Hirudo*** (Shuizhi), *Angelicae sinensis radix* (Danggui), *Moutan cortex* (Mudanpi), *Carthami*		Shang-han-bian-zheng-l

	flos (Honghua), *Glycyrrhizae radix et rhizoma* (Gancao), *Rehmanniae radix* (Dihuang)		u
Shagui Potai Tang	***Hirudo*** (Shuizhi), *Moutan cortex* (Mudanpi), *Angelicae sinensis radix* (Danggui), *Rhei radix et rhizoma* (Gancao), *Magnoliae officinalis cortex* (Houpo), *Carthami flos* (Honghua), *Achyranthis bidentatae radix* (Niuxi), *Rehmanniae radix* (Dihuang), *Persicae semen* (Taoren)		Shang-han-bian-zheng-l u
Shengdihuang Tang	*Rehmanniae radix* (Dihuang), *Toxicodendri resina* (Ganqi), *Lotusroot squeezed juice* (Lian'ou'zhi), *Tabanus* (Mengchong), ***Hirudo*** (Shuizhi), *Rhei radix et rhizoma* (Dahuang)		Lei-zheng-zhi-cai
Taohe Tang	*Eupolyphaga seu steleophaga* (Tubiechong), *Tabanus* (Mengchong), ***Hirudo*** (Shuizhi), *Cinnamomi cortex* (Rougui), *Rhei radix et rhizoma* (Dahuang), *Juglandis semen* (Hetaoren)	Clearing heat and detoxifying	Liu-juan-zi-gui-yi-fang
Taoren Tang	*Persicae semen* (Taoren), *Angelicae sinensis radix* (Danggui), *Trichosanthes cucumeroides radix* (Tuguagen), *Rhei radix et rhizoma* (Dahuang), ***Hirudo*** (Shuizhi), *Tabanus* (Mengchong), *Natrii sulfas* (Mangxiao), *Achyranthis bidentatae radix* (Niuxi), *Cannabis fructus* (Huomaren), *Cinnamomi cortex* (Rougui)	Stimulating menstruation discharge	Qian-jin-yao-fang
Taozhi Tang	*Persicae ramulus* (Taozhi), *Natrii sulfas* (Mangxiao), *Rhei radix et rhizoma* (Dahuang), *Angelicae sinensis radix* (Danggui), *Glycyrrhizae radix et rhizoma* (Gancao), *Cinnamomi cortex* (Rougui), *Tabanus* (Mengchong), ***Hirudo*** (Shuizhi), *Persicae semen* (Taoren)	Treating traumatic injury and blood stasis	Wai-tai-mi-yao
Xierong Tang	*Forsythiae fructus* (Lianqiao), *Cimicifugae rhizome* (Shengma), *Platycodonis radix* (Jiegeng), *Scutellariae radix* (Huangqin), *Rehmanniae radix* (Dihuang), *Astragali radix* (Huangqi), *Sappan lignum* (Sumu), *Coptidis rhizoma* (Huanglian), *Pheretima* (Dilong), *Scorpio* (Quanxie), *Angelicae sinensis radix* (Danggui), *Amomumkravanh pirreex grgnep* (Baidoukou), *Ginseng radix et rhizoma* (Renshen), *Glycyrrhizae radix et rhizoma* (Gancao), *Populi resina* (Wutonglei), *Moschus* (Shexiang), *Persicae semen* (Taoren), *Tabanus* (Mengchong), ***Hirudo*** (Shuizhi)	Clearing heat and detoxifying	Lan-shi-mi-cang

Xingren Tang	*Armeniacae semen amarum* (Kuxingren), *Persicae semen* (Taoren), *Rhei radix et rhizoma* (Dihuang), ***Hirudo*** (Shuizhi), *Tabanus*	Promoting blood circulation and regulating menstrual function	Qian-jin-yao-fang
Yanmi Tang	*Euodiae fructus, Rhei radix et rhizoma, Angelicae sinensis radix, Zingiberis rhizoma, Tabanus,* ***Hirudo*** (Shuizhi), *Rehmanniae radix* (Dihuang), *Chuanxiong rhizoma* (Chuanxiong), *Gardeniae fructua* (Zhizi), *Persicae semen* (Taoren), *Paeoniae radix alba* (Baishao), *Asari radix et rhizome* (Xixin), *Glycyrrhizae radix et rhizoma* (Gancao), *Cinnamomi cortex* (Rougui), *Achyranthis bidentatae radix* (Niuxi), *Cannabis fructus* (Huomaren)	Regulating menstrual function for pain relief	Qian-jin-yi-fang
Yishi Mieguai Tang	*Ginseng radix et rhizoma* (Renshen), *Corni fructus* (Shanzhuyu), *Angelicae sinensis radix* (Danggui), *Olibanum* (Ruxiang), *Tabanus* (Mengchong), ***Hirudo*** (Shuizhi), *Faeces bombycis* (Chansha)		Shang-han-bian-zheng-l u
Zhuyu Mengchong Tang	*Euodiae fructus* (Wuzhuyu), *Tabanus* (Mengchong), ***Hirudo*** (Shuizhi), *Eupolyphaga seu steleophaga* (Tubiechong), *Moutan cortex* (Mudanpi), Fresh ginger (Shengjiang), *Triticum aestivum* (Xiaomai), *Pinelliae rhizome* (Banxia), *Jujubae fructus* (Dazao), *Persicae semen* (Taoren), *Ginseng radix et rhizome* (Renshen), *Achyranthis bidentatae radix* (Niuxi), *Cinnamomi cortex* (Rougui), *Glycyrrhizae radix et rhizoma* (Gancao), *Paeoniae radix alba* (Baishao)	Warming spleen and stomach for regulating menstrual function	Qian-jin-yao-fang
Banmao San	*Mylabris* (Banmao), *Rhei radix et rhizoma* (Dahuang), ***Hirudo*** (Shuizhi), *Angelicae sinensis radix* (Danggui), *Tabanus* (Mengchong)	Simulating menstrual function	Tai-ping-sheng-hui-Fang
Bisheng San	*Halite violaceous* (Naosha), *Draconis sanguis* (Xuejie), *Myrrha* (Moyao), *Hippocampus* (Haima), *Cinnamomi cortex* (Rougui), *Aucklandiae radix* (Muxiang), *Cinnabaris* (Zhusha), *Toxicodendri resina* (Ganqi), *Tabanus* (Mengchong), *Borneolum syntheticum* (Bingpian), ***Hirudo*** (Shuizhi), *Angelicae sinensis radix* (Danggui), *Borax* (Pengsha), *Ferulae resina* (Awei)	Clearing heat and promoting fluid production, stoping bleeding	Ji-feng-pu-ji-fang

Dahuang San	*Rhei radix et rhizoma* (Danggui), *Natrii sulfas* (Mangxiao), *Achyranthis bidentatae radix* (Niuxi), *Angelicae sinensis radix* (Danggui), *Persicae semen* (Taoren), *Tabanus* (Mengchong), *Paeoniae radix rubra* (Chishao), ***Hirudo*** (Shuizhi), *Trichosanthes cucumeroides radix* (Tuguagen), *Toxicodendri resina* (Ganqi), *Cinnamomi cortex* (Rougui)	Regulating menstrual function for pain relief	Tai-ping-sheng-hui-Fang
Daimao San	*Hawksbill* (Daimao), *Corydalis yanhusuo* (yanhusuo), *Angelicae sinensis radix* (Danggui), *Scales carps* (Chilinjia), *Moschus* (Shexiang), *Succinum* (Hupo), ***Hirudo*** (Shuizhi), *Moutan cortex* (Mudanpi), *Typhae pollen* (Puhuang), *Leonuri herba fructus* (Yimucaozi)	Promoting blood circulation and regulating menstrual function	Tai-ping-sheng-hui-Fang
Chilonglin San	*Scales carps* (Chililin), *Tabanus* (Mengchong), ***Hirudo*** (Shuizhi), *Typhae pollen* (Puhuang), *Crinis carbonisatus* (Xueyutan)	Promoting blood circulation for removing stasis	Tai-ping-sheng-hui-Fang
Dilong San	*Pheretima* (Dilong), *Lizards* (Xiyi), *Chuanxiong rhizoma* (Chuanxiong), *Cinnamomi cortex* (Rougui), *Zingiberis rhizoma* (Ganjiang), *Sappan lignum* (Sumu), *Aucklandiae radix* (Muxiang), *Typhae pollen* (Puhuang), *Paeoniae radix rubra* (Chishao), *Moutan cortex* (Mudanpi), ***Hirudo*** (Shuizhi), *Persicae semen* (Taoren)	Removing stasis for pain relief	Tai-ping-sheng-hui-Fang
Guijian San	*Euonymus alatus ramulus* (Guijianyu), *Paeoniae radix rubra* (Chishao), *Rhei radix et rhizoma* (Dahuang), *Cinnamomi cortex* (Rougui), *Trionycis carapax* (Biejia), *Angelicae sinensis radix* (Danggui), *Achyranthis bidentatae radix* (Niuxi), *Succinum* (Hupo), *Trichosanthes cucumeroides radix* (Tuguagen), ***Hirudo*** (Shuizhi), *Natrii sulfas* (Mangxiao), *Polygoni cuspidati rhizoma et radix* (Huzhang), *Persicae semen* (Taoren), *Tabanus* (Mengchong)	Promoting blood circulation and regulating menstrual function	Tai-ping-sheng-hui-Fang
Guixin San	*Cinnamomi cortex* (Rougui), ***Hirudo*** (Shuizhi), *Moutan cortex* (Mudanpi), *Corydalis yanhusuo* (Yanhusuo), *Sulfur* (Liuhuang)	Treating postpartum blood stasis	Tai-ping-sheng-hui-Fang
Hupo San	*Succinum* (Hupo), *Achyranthis bidentatae radix* (Niuxi), *Angelicae sinensis radix* (Danggui), *Corydalis yanhusuo* (Yanhusuo), *Persicae semen* (Taoren), *Chuanxiong rhizoma*	Regulating menstrual function for	Tai-ping-sheng-hui-Fang

	(Chuanxiong)*, Paeoniae radix rubra* (Chishao)*, Cinnamomi cortex* (Rougui)*, Rhei radix et rhizoma* (Dahuang)*, Moutan cortex* (Mudanpi)*,* ***Hirudo*** (Shuizhi)	pain relief	
Huangqin San	*Scutellariae radix* (Huangqin)*, Moutan cortex* (Mudanpi)*, Dianthi herba* (Qumai)*, Paeoniae radix rubra* (Chishao)*, Persicae semen* (Taoren)*, Aurantii fructus immaturus* (Zhishi)*, Chuanxiong rhizoma* (Chuanxiong)*, Belamcandae rhizoma* (Shegan)*, Sargassum* (Haizao)*, Tabanus* (Mengchong)*,* ***Hirudo*** (Shuizhi)*, Holotrichiadiomphalia bates* (Qicao)*, Rhei radix et rhizoma* (Dahuang)	Promoting blood circulation and nourishing blood, simulating menstrual function	Tai-ping-sheng-hui-Fang
Jianghuang San	*Curcumae longae rhizoma* (Jianghuang)*, Moutan cortex* (Mudanpi)*, Angelicae sinensis radix* (Danggui)*, Tabanus* (Mengchong)*, Myrrha* (Moyao)*,* ***Hirudo*** (Shuizhi)*, Artemisia anomala* (Liujinu)*, Cinnamomi cortex* (Rougui)*, Achyranthis bidentatae radix* (Niuxi)	Treating postpartum blood stasis, and pain	Tai-ping-sheng-hui-Fang
Jiegu san	*Scolopendra* (Wugong)*, Pyritum* (Zirantong)*, Olibanum* (Ruxiang)*, Hydrocotylesibthorpioidesvar* (Tongqian)*,* ***Hirudo*** (Shuizhi)*, Myrrha* (Moyao)	Treating traumatic injury, swelling	Ru-men-shi-qin
Jiujiang San	*Angelicae sinensis radix* (Danggui)*, Photinia serrulata* (Shinan)*, Gentianae macrophyllae radix* (Qinjiao)*, Spider* (Zhizhu)*, Chrysanthemi flos* (Juhua)*, zingiberis rhizoma* (Ganjiang)*, Saposhnikoviae radix* (Fangfeng)*, Moschus* (Shexiang)*, Realgar* (Xionghuang)*, Cinnabaris* (Zhusha)*, Mylabris* (Banmao)*, Zanthoxylum bungeanum pericarpium* (Huajiao)*, Forsythiae fructus* (Lianqiao)*, Anemarrhenae rhizoma* (Zhimu)*, Euonymus alatus ramulus* (Guijianyu)*, Aconiti lateralis radix praeparata* (Fuzi)*, Vaccariae semen* (Wangbuliuxing)*, Ginseng radix et rhizoma* (Renshen)*, Podophyllum peltatum* (Guijiu)*, Stephaniae tetrandrae radix* (Fangji)*, Dendrobii caulis* (Shihu)*, Aconiti radix* (Chuanwu)*, Angelicae pubescentis radix* (Duhuo)*, Elephantopus scaber* (Didan)*, Tabanus* (Mengchong)*, Scolopendra* (Wugong)*,* ***Hirudo*** (Shuizhi)	Treating leucoderma	Qian-jin-yi-fang
Longlin San	*Carp skin* (Liyupi)*, Crinis carbonisatus* (Xueyutan)*, Eupolyphaga seu steleophaga*	Clearing heat, cooling blood,	Ji-feng-pu-ji-fang

	(Tubiechong), ***Hirudo*** (Shuizhi), *Squama manitis* (Chuanshanjia), *Gleditsiae abnormalis fructus* (Zhuyazaojiao), *Typhae pollen* (Puhuang), *Draconis sanguis* (Xuejie), *Myrrha* (Moyao), *Moschus* (Shexiang), *Succinum* (Hupo)	promoting blood circulation for removing stasis	
Mudan San	*Moutan cortex* (Mudanpi), *Angelicae sinensis radix* (Danggui), *Angelicae dahuricae radix* (Baizhi), *Succinum* (Hupo), *Rhei radix et rhizoma* (Dahuang), *Paeoniae radix rubra* (Chishao), *Cinnamomi cortex* (Rougui), *Chuanxiong rhizoma* (Chuanxiong), *Tabanus* (Mengchong), ***Hirudo*** (Shuizhi)	Regulating the menstrual function	Tai-ping-sheng-hui-Fang
Poxue San	*Notopterygii rhizoma et radix* (Qianghuo), *Saposhnikoviae radix* (Fangfeng), *Cinnamomi cortex* (Rougui), *Bupleuri radix* (Chaihu), *Angelicae sinensis radix* (Danggui), ***Hirudo*** (Shuizhi), *Moschus* (Shexiang), *Sappan lignum* (Sumu)	Promoting blood circulation, dispelling stasis, pain relief	Yu-ji-wei-yi
Rougui San	*Cinnamomi cortex* (Rougui), *Angelicae sinensis radix* (Danggui), *Typhae pollen* (Puhuang), *Achyranthis bidentatae radix* (Niuxi), *Euonymus alatus ramulus* (Guijianyu), *Tabanus* (Mengchong), *Succinum* (Hupo), *Paeoniae radix rubra* (Chishao), *Persicae semen* (Taoren), ***Hirudo*** (Shuizhi), *Rhei radix et rhizoma* (Dahuang)	Stoping bleeding, promoting blood circulation, dispelling stasis	Tai-ping-sheng-hui-Fang
Shenxiao Jiegu San	***Hirudo*** (Shuizhi), *Os tigris* (Hugu), *Testudinis carapax et plastrum* (Guijia), *Pyritum* (Zirantong), *Momordicae semen* (Mubiezi), *Pharbitidis semen* (Heiqianniu), *Tetrapanacis medulla* (Tongcao), *Olibanum* (Ruxiang), *Myrrha* (Moyao), *Vignae semen* (Chixiaodou), *Angelicae sinensis radix* (Danggui), *Saposhnikoviae radix* (Fangfeng), *Linderae radix* (Wuyao), *Aconiti kusnezoffii radix* (Caowu)	Promoting blood circulation, activating Qi, relieving pain	Feng-ke-ji-yan-ming-fan g
Shuizhi San	***Hirudo*** (Shuizhi),*Tabanus* (Mengchong), *Achyranthis bidentatae radix* (Niuxi), *Moutan cortex* (Mudanpi), *Persicae semen* (Taoren), *Cinnamomi cortex* (Mudanpi), *Artemisia keiskeana fructus* (Anzi), *Angelicae sinensis radix* (Danggui), *Trionycis carapax* (Biejia), *Toxicodendri resina* (Ganqi), *Euonymus alatus ramulus* (Guijianyu), *Succinum* (Hupo),	Promoting blood circulation, eliminating stasis, relieving pain	Tai-ping-sheng-hui-Fang

	Euodiae fructus (Wuzhuyu), *Genkwa flos* (Yuanhua), *Moschus* (Shexiang)		
Taoren San	*Persicae semen* (Taoren), *Rhei radix et rhizoma* (Dahuang), *Tabanus* (Mengchong), ***Hirudo*** (Shuizhi), *Natrii sulfas* (Mangxiao), *Cinnamomi cortex* (Rougui), *Angelicae sinensis radix* (Danggui), *Glycyrrhizae radix et rhizoma* (Gancao)	Promoting blood circulation, eliminating stasis	Tai-ping-sheng-hui-Fang
Wujin San	*Carp skin carbonisatus* (Chililintan), *Crinis carbonisatus* (Xueyutan), *Terra flava usta* (Fulonggan), *Porcinefat* (Layuezhuzhi), ***Hirudo*** (Shuizhi), *Cinnamomi cortex* (Rougui), *Angelicae sinensis radix* (Danggui), *Moschus* (Shexiang)	Warming spleen and stomach for removing stasis	Tai-ping-sheng-hui-Fang
Xingren San	*Armeniacae semen amarum* (Kuxingren), *Rhei radix et rhizoma* (Dahuang), ***Hirudo*** (Shuizhi), *Tabanus* (Mengchong), *Persicae semen* (Taoren)	Treating irregular menses	Tai-ping-sheng-hui-Fang
Xionghuang San	*Realgar* (Xionghuang), *Halite violaceous* (Naosha), *Moschus* (Shexiang), *Felursi* (Xiongdan), ***Hirudo*** (Shuizhi)	Eliminating stasis, relieving pain	Tai-ping-sheng-hui-Fang
Xuejie San	*Halite violaceous* (Naosha), *Draconis sanguis* (Xuejie), *Myrrha* (Moyao), *Cinnamomi cortex* (Rougui), *Aucklandiae radix* (Muxiang), *Cinnabaris* (Zhusha), *Hippocampus* (Haima), *Toxicodendri resina* (Ganqi), *Tabanus* (Mengchong), *Borneolum syntheticum* (Bingpian), ***Hirudo*** (Shuizhi), *Angelicae sinensis radix* (Danggui), *Borax* (Pengsha), *Ferulae resina* (Awei)	Clearing heat to stop bleeding, thirst relief	Ji-feng-pu-ji-fang
Yimucaozi San	*Leonuri herba fructus* (Yimucaozi), *Cinnamomi cortex* (Rougui), *Angelicae sinensis radix* (Danggui), *Paeoniae radix rubra* (Chishao), *Rehmanniae radix praeparata* (Shudihuang), *Hordei fructus Germinatus* (Maiya), *Euonymus alatus ramulus* (Guijianyu), *Rhei radix et rhizoma* (Dahuang), *Carp skin carbonisatus* (Chililintan), *Crinis carbonisatus* (Xueyutan), *Lithargyrum* (Mituoseng), *Tabanus* (Mengchong), ***Hirudo*** (Shuizhi), *Moschus* (Shexiang)	Promoting blood circulation, simulating menstrual function, nourishing blood for pain relief	Tai-ping-sheng-hui-Fang
Yuanhua San	*Genkwa flos* (Yuanhua), *Aconiti radix* (Chuanxiong), *Euonymus alatus ramulus* (Guijianyu), *Tabanus* (Mengchong), ***Hirudo*** (Shuizhi), *Persicae semen* (Taoren)	Eliminating stasis, relieving pain	Tai-ping-sheng-hui-Fang

Yuntaizi San	*Yuntaizi, Rhei radix et rhizoma* (Dahuang), *Myrrha* (Moyao), *Typhae pollen* (Puhuang), ***Hirudo*** (Shuizhi), *Calomelas* (Qingfen), *Rehmanniae radix* (Dihuang), ginger juice	Promoting blood and eliminating stasis, dispelling tumefaction	Tai-ping-sheng-hui-Fang
Guixin Jiu	*Cinnamomi cortex* (Rougui), *Moutan cortex* (Mudanpi), *Paeoniae radix alba* (Baishao), *Achyranthis bidentatae radix* (Niuxi), *Toxicodendri resina* (Ganqi), *Trichosanthes cucumeroides radix* (Tuguagen), *Euodiae fructus* (Wuzhuyu), *Rhei radix et rhizoma* (Dahuang), *Scutellariae radix* (Huangqin), *Zingiberis rhizoma* (Ganjiang), *Tabanus* (Mengchong), *Eupolyphaga seu steleophaga* (Tubiechong), *Holotrichiadiomphalia bates* (Qicao), ***Hirudo*** (Shuizhi),*Crinis carbonisatus* (Xueyutan), *Asari radix et rhizoma* (Xixin), *Bombyx batryticatus* (Jiangchan), *Cannabis fructus* (Huomaren), *Rehmanniae radix* (Dihuang), *Polygoni cuspidati rhizoma et radix* (Huzhang), *Trionycis carapax* (Biejia), *Artemisia keiskeana fructus* (Anzi)	Eliminating blood stasis and simulating menstrual function	Qian-jin-yao-fang
Huazheng Dan	*Ginseng radix et rhizoma* (Renshen), *Cinnamomi cortex* (Rougui), *Anemones raddeanae rhizoma* (Liangtoujian), ***Hirudo*** (Shuizhi), *Wenyujin rhizoma concisum* (Pianjianghuang), *Eugenia caryophyllata* Thunb (Gongdingxiang), *Zanthoxylum bungeanum carbonisatus* (Huajiaotan), *Tabanus* (Mengchong), *Sparganii rhizoma* (Sanleng), *Typhae pollen* (Puhuangtan), *Moschus* (Shexiang), *Sappan lignum* (Sumu), *Persicae semen* (Taoren), *Perillae fructus* (processed, Suzishuang), *Faeces trogopterori* (Wulingzhi), *Dalbergiae odoriferae lignum* (Jiangxiang), *Toxicodendri resina* (Ganqi), *Myrrha* (Moyao), *Paeoniae radix alba* (Baishao), *Armeniacae semen amarum* (Kuxingren), *Cyperi rhizoma* (Xiangfu), *Euodiae fructus* (Wuzhuyu), *Corydalis yanhusuo* (Yanhusuo), *Angelicae sinensis radix* (Danggui), *Ferulae resina* (Awei), *Foeniculi fructus carbonisatus* (Xiaohuixiangtan), *Chuanxiong rhizoma* (Chuanxiong), *Olibanum* (ruxiang), *Alpiniae officinarum rhizoma* (Gaoliangjiang), *Artemisiae argyi carbonisatus* (Aitan), *Leonurus liquid extract* (Yimucaogao), *Rehmanniae radix praeparata* (Shudihuang), *Trionycis carapax* (Biejia), *Rhei radix et rhizoma* (Dahuang)	Nourishing blood and activating blood, eliminating blood stasis and simulating menstrual function, relieving pain and treating furuncle	Lin-zheng-jing-yan-lu

Huazheng Huisheng Dan	*Trionycis carapax* (Biejia), *Ginseng radix et rhizoma* (Renshen), *Persicae semen* (Taoren), *Leonurus liquid extract* (Yimugao), *Rehmanniae radix praeparata* (Shudihuang), *Carthami flos* (Honghua), *Eugenia caryophyllata* Thunb (Gongdingxiang), *Paeoniae radix alba* (Baishao), *Moschus* (Shexiang), *Foeniculi fructus carbonisatus* (Xiaohuixiangtan), *Angelicae sinensis radix* (Danggui), *Toxicodendri resina* (Ganqi), *Faeces trogopterori* (Wulingzhi), *Armeniacae semen amarum* (Kuxingren), *Chuanxiong rhizoma* (Chuanxiong), *Sparganii rhizoma* (Sanleng), *Sappan lignum* (Sumu), *Cyperi rhizoma* (Xiangfu), *Perillae fructus* (processed, Suzishuang), *Cinnamomi cortex* (Rougui), *Ferulae resina* (Awei), *Corydalis yanhusuo* (Yanhusuo), *Dalbergiae odoriferae lignum* (Jiangxiang), *Artemisiae argyi carbonisatus* (Aitan), *Wenyujin rhizoma concisum* (Pianjianghuang), *Euodiae fructus* (Wuzhuyu), *Alpiniae officinarum rhizoma* (Gaoliangjiang), *Anemones raddeanae rhizoma* (Liangtoujian), *Olibanum* (Ruxiang), ***Hirudo*** (Shuizhi), *Zanthoxylum bungeanum carbonisatus* (Huajiaotan), *Myrrha* (Moyao), *Tabanus* (Mengchong), *Typhae pollen* (Puhuangtan), *Rhei radix et rhizoma* (Dahuang)	Nourishing blood and activating blood, eliminating blood stasis and simulating menstrual function, relieving pain and treating furuncle	Wu-ju-tong-yi-an
Yangyin Gao	*Rehmanniae radix* (Dihuang), *Angelicae sinensis radix* (Danggui), *Paeoniae radix rubra* (Chishao), *Achyranthis bidentatae radix* (Niuxi), *Linderae radix* (Wuyao), *Moutan cortex* (Mudanpi), *Poria* (Fuling), *Carthami flos* (Honghua), ***Hirudo*** (Shuizhi)	Activating blood and nourishing blood, simulating menstrual function	Ji-feng-pu-ji-fang

Table S2 The patented drugs containing Shuizhi and their clinical uses (222 Drug approval number)

Preparation name	Main compositions	Clinical uses	References
Naoxuekang Pill, Tablet, Oral Liquid, Granule, Capsule, drippling Pill	***Hirudo*** (Shuizhi)	Stroke and intracerebral hemorrhage	[5]
Huoxue Tongmai Capsule	***Hirudo*** (Shuizhi)	Amenorrhea with syndrome of blood stasis and hyperlipemia	[5]
Compound Maqitong Capsule	***Hirudo*** (Shuizhi), *Eupolyphaga* or *Steleophaga* (Tubeichong)	Stable coronary disease and angina	[5]

Zhongfeng'an Oral Liquid	***Hirudo*** (Shuizhi), *Astragali Radix* (Huangqi)	Acute cerebral thrombosis with syndrome of Qi deficiency and blood stasis	[5]
Shuxuetong Injection	***Hirudo*** (Shuizhi), *Pheretima* (Dilong)	Acute stroke with blood stasis	[5]
Shenyuan Capsule	*Leonuri Herba* (Yimucao), *Polygala Herba* (Guazijin), ***Hirudo*** (Shuizhi)	Edema induced by blood stasis and chronic nephritis	[5]
Zhuyu Tongmai Capsule	***Hirudo*** (Shuizhi), *Persicae semen* (Taoren), *Tabanus* (Mengchong), *Rhei radix et rhizoma* (Dahuang)	Vertigo with blood stasis	[5]
Naosai'an Capsule	***Hirudo*** (Shuizhi), *Tabanus* (Mengchong), *Rhei radix et rhizoma* (Dahuang), *Persicae semen* (Taoren)	Thrombotic cerebral embolism recovery stage	[5]
Wuwei Tongshuan Oral Liquid	*Astragali Radix* (Huangqi), ***Hirudo*** (Shuizhi), *Chuanxiong Rhizoma* (Chuanxiong), *Angelicae sinensis radix* (Danggui), *Salviae miltiorrhizae radix et rhizoma* (Danshen)	Acute cerebral infarction	[5]
Compound Danzhi Tablet	*Astragali Radix* (Huangqi), *Salviae miltiorrhizae radix et rhizoma* (Danshen), ***Hirudo*** (Shuizhi), *Pheretima* (Dilong), *Chuanxiong Rhizoma* (Chuanxiong)	Cerebral infarction recovery stage	[5]
Lyophilizated Naoxinkang powder for injection	*Ginseng Radix et Rhizoma* (Renshen), *Astragali Radix* (Huangqi), *Ganoderma* (Lingzhi), ***Hirudo*** (Shuizhi)	Cerebral infarction, early coronary disease and angina	[5]
Qizhi Jiangtang Capsule	*Astragali Radix* (Huangqi), *Rehmanniae Radix* (Dihuang), *Polygonati Rhizoma* (Huangjing), ***Hirudo*** (Shuizhi)	Type II diabetes and related complications	[5]
Huisheng Oral Liquid	*Leonuri Herba* (Yimucao), *Carthami Flos* (Honghua), *Zanthoxylum bungeanum carbonisatus* (Huajiao Tan), ***Hirudo*** (Shuizhi, processed), *Angelicae sinensis radix*	For the treatment of the primary liver carcinoma and	[5]

	(Danggui), *Sappan lignum* (Sumu), *Sparganii rhizoma* (Sanleng, processed with vinegar), *Anemones Raddeanae Rhizoma* (Liangtoujian), *Chuanxiong rhizoma* (Chuanxiong), *Dalbergiae odoriferae lignum* (Jiangxiang), *Cyperi rhizoma* (Xiangfu, processed with vinegar), *Ginseng Radix et Rhizoma* (Renshen), *Alpiniae officinarum rhizoma* (Gaoliangjiang), *Curcumae longae rhizoma* (Jianghuang), *Myrrha* (Moyao, processed with vinegar), *Armeniacae semen amarum* (Kuxingren, fried), *Rhei radix et rhizoma* (Dahuang), *Perillae Fructus* (Zisuzi), *Foeniculi Fructus* (Xiaohuixiang, fried with salt), *Persicae semen* (Taoren), *Faeces Trogopterori* (Wulingzhi, processed with vinegar), *Tabanus* (Mengchong), *Trionycis carapax* (Biejia), *Caryophylli flos* (Dingxiang), *Corydalis Yanhusuo* (Yanhusuo, processed with vinegar), *Paeoniae radix alba* (Baishao), *Typhae pollen carbonisatus* (Puhuang Tan), *Olibanum* (Ruxiang, processed with vinegar), *Toxicodendri resina* (Ganqi, calcined), *Euodiae fructus* (Wuzhuyu, processed with aqueous extract of *Glycyrrhizae Radix et Rhizoma*), *Ferulae resina* (Awei), *Cinnamomi cortex* (Rougui), *Artemisiae argyi folium* (Aiye, processed), *Rehmanniae radix praeparata* (Shudihuang)	lung carcinoma	
Tongmai Jiangtang Capsule	*Pseudostellariae radix* (Taizishen), *Salviae miltiorrhizae radix et rhizome* (Danshen), *Coptidis rhizoma* (Huanglian), *Astragali radix* (Huangqi), *Gynostemma pentaphyllum* (Jiaogulan), *Dioscoreae rhizome* (Shanyao), *Atractylodis rhizome* (Cangzhu), *Scrophulariae radix* (Xuanshen), ***Hirudo*** (Shuizhi), *Malvae fructus* (Dongkuiguo), *Puerariae lobatae radix* (Gegen)	Replenishing Yin, clearing heat and promoting blood circulation. For the treatment of diabetes	[5]
Jiangtang Tongmai Capsule	*Pseudostellariae radix* (Taizishen), *Astragali radix* (Huangqi), *Polygonati rhizoma* (Huangjing), *Asparagi radix* (Tiandong), *Ophiopogonis radix* (Maidong), *Scrophulariae radix* (Xuanshen), *Trichosanthis Radix* (Tianhuafen), *Atractylodis rhizome* (Cangzhu), *Anemarrhenae rhizome* (Zhimu), *Puerariae lobatae radix* (Gegen), *Coptidis rhizome* (Huanglian), *Salviae miltiorrhizae radix et rhizome* (Danshen), *Leonuri Herba*	Tonifying Qi and Yin, promoting blood circulation and removing stasis. For the treatment of type II diabetes	[5]

	(Yimucao), *Paeoniae radix rubra* (Chishao), ***Hirudo*** (Shuizhi), *Cyathulae radix* (Chuanniuxi), *Spatholobi caulis* (Jixueteng), *Clematidis radix et rhizoma* (Weilingxian), *Litchi semen* (Lizhihe), *Pheretima* (Dilong), *Chuanxiong rhizome* (Chuanxiong)		
Digu Jiangtang Capsule	*Curcumae radix* (Yujin), *Lycii cortex* (Digupi), *Perillae Fructus* (Zisuzi), *Trionycis carapax* (Biejia, processed), *Pheretima* (Dilong), ***Hirudo*** (Shuizhi), *Cordyceps* (Dongchongxiacao)	Diabetes induced by Yin deficiency and blood stasis	[5]
Nao Xin Tong Capsule	*Astragali radix* (Huangqi), *Paeoniae radix rubra* (Chishao), *Salviae miltiorrhizae radix et rhizome* (Danshen), *Angelicae sinensis radix* (Danggui), *Chuanxiong rhizome* (Chuanxiong), *Persicae semen* (Taoren), *Carthami Flos* (Honghua), *Olibanum* (Ruxiang, processed), Myrrha (Moyao, processed), *Spatholobi caulis* (Jixueteng), *Achyranthis bidentatae radix* (Niuxi), *Cinnamomi ramulus* (Guizhi), *Mori ramulus* (Sangzhi), *Pheretima* (Dilong), *Scorpio* (Quanxie), ***Hirudo*** (Shuizhi)	Stroke induced by Qi deficiency and blood stasis	[5]
Naoshuan Kangfu Capsule	*Notoginseng radix et rhizoma* (Sanqi), *Puerariae lobatae radix* (Gegen), *Paeoniae radix rubra* (Chishao), *Carthami Flos* (Honghua), *Siegesbeckiae herba* (Xixiancao), *Draconis Sanguis* (Xuejie), *Chuanxiong rhizome* (Chuanxiong), *Pheretima* (Dilong), ***Hirudo*** (Shuizhi), *Achyranthis bidentatae radix* (Niuxi)	Stroke induced by blood stasis	[5]
Shexiang Xinnao Tong Capsule	*Salviae miltiorrhizae radix et rhizome* (Danshen), *Notoginseng radix et rhizoma* (Sanqi), *Carthami Flos* (Honghua), *Epimedii folium* (Yinyanghuo), *Puerariae lobatae radix* (Gegen), *Curcumae radix* (Yujin), *Persicae semen* (Taoren), *Chuanxiong rhizome* (Chuanxiong), ***Hirudo*** (Shuizhi), *Borneolum Syntheticum* (Bingpian), *Moschus* (Shexiang), Total ginsenoside of ginseng stems and leaves (Renshen Jingye Zongzaogan)	Stroke induced by blood stasis	[5]

Dahuang Zhechong Pill, Capsule	*Rhei radix et rhizoma Praeparata* (Shudahuang), *Eupolyphaga* or *Steleophaga* (Tubeichong, fried), ***Hirudo*** (Shuizhi, processed), *Tabanus* (Mengchong, fried), *Holotrichiadiomphalia Bates* (Qicao, fried), *Toxicodendri resina* (Ganqi, calcined), *Persicae semen* (Taoren), *Armeniacae semen amarum* (Kuxingren, fried), *Scutellariae radix* (Huangqin), *Rehmanniae radix* (Dihuang), *Paeoniae radix alba* (Baishao), *Glycyrrhizae radix et rhizoma* (Gancao)	Invigorating blood, removing blood stasis. For the treatment of hepatic diseases and gynecopathy in clinic practice and occasionally for atherosclerotic therapy	[5] [13]
Huazheng Huisheng Tablet	*Leonuri Herba* (Yimucao), *Carthami Flos* (Honghua), *Zanthoxylum bungeanum carbonisatus* (Huajiao Tan), ***Hirudo*** (Shuizhi, processed), *Angelicae sinensis radix* (Danggui), *Sappan lignum* (Sumu), *Sparganii rhizoma* (Sanleng, processed with vinegar), *Anemones Raddeanae Rhizoma* (Liangtoujian), *Chuanxiong rhizoma* (Chuanxiong), *Dalbergiae odoriferae lignum* (Jiangxiang), *Cyperi rhizoma* (Xiangfu, processed with vinegar), *Ginseng Radix et Rhizoma* (Renshen), *Alpiniae officinarum rhizoma* (Gaoliangjiang), *Curcumae longae rhizoma* (Jianghuang), *Myrrha* (Moyao, processed with vinegar), *Armeniacae semen amarum* (Kuxingren, fried), *Rhei radix et rhizoma* (Dahuang), *Moschus artifactus* (Rengong Shexiang), *Foeniculi Fructus* (Xiaohuixiang, fried with salt), *Persicae semen* (Taoren), *Faeces Trogopterori* (Wulingzhi, processed with vinegar), *Tabanus* (Mengchong), *Trionycis carapax* (Biejia), *Caryophylli flos* (Dingxiang), *Corydalis Yanhusuo* (Yanhusuo, processed with vinegar), *Paeoniae radix alba* (Baishao), *Typhae pollen carbonisatus* (Puhuang Tan), *Olibanum* (Ruxiang, processed with vinegar), *Toxicodendri resina* (Ganqi, calcined), *Euodiae fructus* (Wuzhuyu, processed with aqueous extract of *Glycyrrhizae Radix et Rhizoma*), *Ferulae resina* (Awei), *Cinnamomi cortex* (Rougui), *Artemisiae argyi folium* (Aiye, processed with vinegar), *Rehmanniae radix praeparata* (Shudihuang), *Perillae Fructus* (Zisuzi)	For the gynecopathy diseases, primary liver cancer and lung cancer	[13]
Xueshuan Xinmaining	*Chuanxiong rhizome* (Chuanxiong), *Sophorae flos* (Huai hua), *Salviae miltiorrhizae*	Cerebral thrombosis,	[13]

Tablet, Capsule	*radix et rhizome* (Danshen), ***Hirudo*** (Shuizhi), *Ilex pubescens* (Maodongqing), *Bovis calculus* (Niuhuang), *Moschus* (Shexiang), Total ginsenoside of ginseng stems and leaves (Renshen Jingye Zongzaogan), *Borneolum Syntheticum* (Bingpian), Toad venom (Chansu)	coronary disease and angina pectoris induced by Qi-stagnancy and blood stasis	
Qingnao Jiangya Tablet, Capsule, Granule	*Scutellariae radix* (Huangqin), *Pruneliae spica* (Xiakucao), *Sophorae flos* (Huai hua), Magnetitum (Cishi, Calcined), *Achyranthis bidentatae radix* (Niuxi), *Angelicae sinensis radix* (Danggui), *Rehmanniae radix* (Dihuang), *Salviae miltiorrhizae radix et rhizoma* (Danshen), ***Hirudo*** (Shuizhi), *Uncariae ramulus cum unicis* (Gouteng), Cassiae semen (Juemingzi), *Pheretima* (Dilong), *Margaritifera concha* (Zhenzhumu)	Vertigo induced by hyperactivity of liver-YANG, with the symptoms of dizziness, headache, stiff neck and hypertension	[13]
Kangshuan Zaizao Pill	*Ginseng radix et rhizome rubra* (Hongshen), *Astragali radix* (Huangqi), *Arisaematis rhizoma* (Dannanxing, processed with bile), *Manis squama* (Chuanshanjia, processed), *Bovis calculus* (Niuhuang), *Borneolum Syntheticum* (Bingpian), ***Hirudo*** (Shuizhi, processed), *Moschus* (Shexiang), *Salviae miltiorrhizae radix et rhizoma* (Danshen), *Notoginseng radix et rhizoma* (Sanqi), *Rhei radix et rhizoma* (Dahuang), *Pheretima* (Dilong), Styrax (Suhexiang), *Scorpio* (Quanxie), *Puerariae lobatae radix* (Gegen), *Dioscoreae nipponicae rhizoma* (Chuanshanlong), *Angelicae sinensis radix* (Danggui), *Achyranthis bidentatae radix* (Niuxi), *Polygoni multiflori radix* (Heshouwu), *Zaocys* (Wushaoshe), *Persicae semen* (Taoren), *Cinnabaris* (Zhusha), *Carthami Flos* (Honghua), *Eupolyphaga* or *Steleophaga* (Tubeichong), *Gastrodiae rhizoma* (Tianma), *Asari radix et rhizoma* (Xixin), *Clematidis radix et rhizoma* (Weilingxian), *Alpiniae katsumadai semen* (Caodoukou), G*lycyrrhizae radix et rhizoma* (Gancao)	Cerebral infarction recovery stage	[14]
Nangchong Pill	*Poria* (Fuling), ***Hirudo*** (Shuizhi, processed), *Omphalia* (Leiwan), *Rhei radix et rhizoma* (Dahuang), *Bombyx batryticatus* (Jiangchan, fried), *Persicae semen* (Taoren), *Coptidis rhizome* (Huanglian), *Moutan cortex* (Mudanpi), *Aconiti radix* (Chuanwu), Genkwa	cysticercosis, cerebral cysticercosis and their	[14]

	flos (processed with vinegar), *Citri grandis exocarpium* (Huajuhong), *Faeces trogopterori* extract (Wulingzhi Liujingao)	related epilepsy	
Shexiang kangshuan Pill, Capsule	*Moschus artifactus* (Rengong Shexiang), *Saigae Tataricae Cornu* (Lingyangjiao), *Scorpio* (Quanxie), *Zaocys* (Wushaoshe), *Notoginseng radix et rhizoma* (Sanqi), *Bombyx batryticatus* (Jiangchan), ***Hirudo*** (Shuizhi, processed), *Chuanxiong rhizome* (Chuanxiong), *Gastrodiae rhizoma* (Tianma), *Rhei radix et rhizoma* (Dahuang), *Carthami Flos* (Honghua), *Arisaematis rhizoma* (Dannanxing, processed with bile), *Spatholobi caulis* (Jixueteng), *Paeoniae radix rubra* (Chishao), *Puerariae lobatae radix* (Gegen), *Rehmanniae radix* (Dihuang), *Astragali radix* (Huangqi), *Lonicerae japonicae caulis* (Rendongteng), *Angelicae sinensis radix* (Danggui), *Trachelospermi caulis et folium* (Luoshiteng), *Pheretima* (Dilong), *Siegesbeckiae herba* (Xixiancao)	Stroke, hemiplegia, alalia, and dizziness	[13]<br>[14]
Naosaitong Pill	*Toxicodendri resina carbonisatus* (Ganqi Tan), *Ginseng radix et rhizome rubra* (Hongshen), *Astragali radix* (Huangqi), *Achyranthis bidentatae radix* (Niuxi), *Trichosanthis Radix* (Tianhuafen), *Eupolyphaga* or *Steleophaga* (Tubeichong, fried), *Moutan cortex* (Mudanpi), *Rhei radix et rhizoma praeparata* (Zhidahuang), *Euodiae fructus* (Wuzhuyu, processed with salt), *Persicae semen* (Taoren), *Natrii sulfas* powder (Xuanmingfen), *Chuanxiong rhizome* (Chuanxiong), *Descurainiae semen* (Tinglizi), *Pheretima* (Dilong, fried), *Orobanche coerulescens herba* (Liedang, processed with wine), *Rehmanniae radix* (Dihuang), ***Hirudo*** (Shuizhi, processed), *Cinnamomi cortex* (Rougui), *Poria* (Fuling), *Succinum* (Hupo), *Cinnabaris* (Zhusha)	Cerebral thrombosis, sequelae of cerebral blood stasis, limb paralysis, numbness and language barrier	[14]
Naoxueshuan Tablet	*Carthami Flos* (Honghua), *Angelicae sinensis radix* (Danggui), ***Hirudo*** (Shuizhi, processed), *Paeoniae radix rubra* (Chishao), *Persicae semen* (Taoren), *Chuanxiong rhizome* (Chuanxiong), *Salviae miltiorrhizae radix et rhizome* (Danshen), *Eupolyphaga* or *Steleophaga* (Tubeichong), *Saigae Tataricae Cornu* (Lingyangjiao),	Stroke aura induced by blood stasis and hyperactivity of liver-Yang	[14]

	Bovis calculus (Niuhuang)		
Loulian Capsule	*Hedyotis diffusa herba* (Baihuashe shecao), *Semiaquilegiae radix* (Tiankuizi), *Polygoni orientalis frucuts* (Shuihonghuazi), *Paridis rhizoma* (Chonglou), *Trionycis carapax* (Biejia, processed), *Curcumae rhizoma* (Ezhu), *Lobeliae chinensis herba* (Banbianlian), *Eupolyphaga* or *Steleophaga* (Tubeichong), ***Hirudo*** (Shuizhi, processed), *Ginseng radix et rhizome rubra* (Hongshen), *Polygoni multiflori radix Praeparata* (Zhi Heshouwu), *Solanum nigrum* L (Longkui)	Auxiliary drugs for primary liver cancer	[5]
Peiyuan Tongnao Capsule	*Polygoni multiflori radix Praeparata* (Zhi Heshouwu), *Rehmanniae radix praeparata* (Shudihuang), *Asparagi radix* (Tiandong), *Trionycis carapax* (Biejia, processed with vinegar), *Cervi cornu pantotrichum* (Lurong), *Cistanches herba* (Roucongrong, processed with wine), *Cinnamomi cortex* (Rougui), *Paeoniae radix rubra* (Chishao), *Scorpio* (Quanxie), ***Hirudo*** (Shuizhi, processed), *Pheretima* (Dilong), *Crataegi frucuts* (Shanzha, fried)	Ischemic stroke	[5]
Xizhi Luoda Capsule	*Siegesbeckiae herba* (Xixiancao, processed with honey), ***Hirudo*** (Shuizhi), *Pinelliae rhizoma* (Banxia, processed with ginger), Gentianae macrophyliae radix (Qinjiao), *Gastrodiae rhizoma* (Tianma), *Eupolyphaga* or *Steleophaga* (Tubeichong), *Notoginseng radix et rhizoma* (Sanqi), *Chuanxiong rhizome* (Chuanxiong), *Carthami Flos* (Honghua), *Borneolum Syntheticum* (Bingpian)	Ischemic stroke	[5]
Anluo Huaxian Pill	*Rehmanniae radix* (Dihuang), *Notoginseng radix et rhizoma* (Sanqi), ***Hirudo*** (Shuizhi), *Bombyx batryticatus* (Jiangchan), *Atractylodis macrocephalae rhizoma* (Baizhu), *Curcumae radix* (Yujin), *Bovis calculus* (Niuhuang), *Arcae concha* (Walengzi), *Rhei radix et rhizoma* (Dahuang), *Hordei fructus germinatus* (Maiya), *Galli gigerii endothelium corneum* (Jineijin), Powdered *buffalo horn* extract (Shuiniujiao	Chronic hepatitis B	[5]

	Nongsuofen), *Pheretima* (Dilong), *Moutan cortex* (Mudanpi)		
Danqi Piantan Capsule	*Astragali radix* (Huangqi), *Salviae miltiorrhizae radix et rhizome* (Danshen), *Paeoniae radix rubra* (Chishao), *Chuanxiong rhizome* (Chuanxiong), *Angelicae sinensis radix* (Danggui), *Carthami Flos* (Honghua), ***Hirudo*** (Shuizhi), *Eupolyphaga* or *Steleophaga* (Tubeichong), *Persicae semen* (Taoren), *Bovis calculus artifactus* (Rengong Niuhuang) *Saigae Tataricae Cornu* (Lingyangjiao), *Scorpio* (Quanxie), *Polygalae radix* (Yuanzhi), *Acori tatarinowii rhizoma* (Shichangpu)	Cerebral infarction recovery stage	[5]
Duzhi Pill	*Eucommiae Cortex* (Duzhong, processed with salt), *Angelicae sinensis radix* (Danggui), *Rehmanniae radix* (Dihuang), *Morindae officinalis radix* (Bajitian), ***Hirudo*** (Shuizhi, processed), *Cynanchi Atrati Radix et Rhizoma* (Baiwei), *Epimedii folium* (Yinyanghuo), *Paeoniae radix rubra* (Chishao), *Acori tatarinowii rhizoma* (Shichangpu), *Astragali radix* (Huangqi), *Leonuri Herba* (Yimucao), *Lycopodii herba* (Shenjincao)	Cerebral infarction recovery stage	[5]
Huangzhi Yishen Capsule	*Astragali radix* (Huangqi), *Lycii fructus* (Gouqizi), *Dioscoreae rhizome* (Shanyao), *Coicis semen* (Yiyiren), *Scrophulariae radix* (Xuanshen), *Glehniae radix* (Beishashen), *Ecliptae herba* (Mohanlian), *Hominis placenta* (Ziheche), *Eucommiae Cortex* (Duzhong), *Notoginseng radix et rhizoma* (Sanqi), *Leonuri Herba* (Yimucao), ***Hirudo*** (Shuizhi), *Cicadae periostracum* (Chantui), *Plantaginis semen* (Cheqianzi), *Achyranthis bidentatae radix* (Niuxi)	Nephritis induced by Qi-Yin deficiency or blood stasis	[5]
Longshengzhi Capsule	*Astragali radix* (Huangqi), ***Hirudo*** (Shuizhi), *Chuanxiong rhizome* (Chuanxiong), *Angelicae sinensis radix* (Danggui), *Carthami Flos* (Honghua), *Paeoniae radix rubra* (Chishao), *Aucklandiae radix* (Muxiang), *Acori tatarinowii rhizoma* (Shichangpu), *Pheretima* (Dilong), *Taxilli herba* (Sangjisheng), *Persicae semen* (Taoren),	Cerebral infarction induced by arterialsclerosis	[5]

	Acanthopanacis senticosi radix et rhizome seu caulis extract (Ciwujia extract)		
Tiaozhi Tablet	*Polygoni multiflori radix Praeparata* (Zhi Heshouwu), *Cassiae semen* (Juemingzi), *Artemisiae scopariae herba* (Yinchen), ***Hirudo*** (Shuizhi), *Crataegi frucuts* (Shanzha), *Rhei radix et rhizoma* (Dahuang, processed with wine), *Curcumae radix* (Yujin)	Primary hyperlipemia	[5]
Lishui Tiaozhi Capsule	*Pinelliae rhizoma* (Fabanxia, processed), *Citri reticulatae pericarpium* (Chenpi), *Chuanxiong rhizome* (Chuanxiong), ***Hirudo*** (Shuizhi)500g *Poria* (Fuling), *Glycyrrhizae radix et rhizoma* (Gancao), bamboo juice	Hyperlipemia	[5]
Qishen Capsule	*Astragali radix* (Huangqi), *Ginseng Radix et Rhizoma* (Renshen), *Notoginseng radix et rhizoma* (Sanqi), *Carthami Flos* (Honghua), *Crataegi frucuts* (Shanzha), *Polygoni multiflori radix Praeparata* (Zhi Heshouwu), *Scutellariae radix* (Huangqin), *Glycyrrhizae radix et rhizoma* (Gancao), *Salviae miltiorrhizae radix et rhizome* (Danshen), *Poria* (Fuling), ***Hirudo*** (Shuizhi), *Chuanxiong rhizoma* (Chuanxiong), *Typhae pollen* (Puhuang), *Puerariae lobatae radix* (Gegen), *Scrophulariae radix* (Xuanshen)	Benefiting Qi and promoting blood circulation, removing stasis and relieving pain. For the treatment of coronary disease and angina	[13]
Tongxinluo Tablet, Capsule	*Ginseng Radix et Rhizoma* (Renshen), ***Hirudo*** (Shuizhi), *Scorpio* (Quanxie), *Paeoniae radix rubra* (Chishao), *Cicadae periostracum* (Chantui), *Eupolyphaga* or *Steleophaga* (Tubeichong), *Scolopendra* (Wugong), *Santali albi lignum* (Tanxiang), *Dalbergiae odoriferae lignum* (Jiangxiang), *Olibanum* (Ruxiang, processed), *Ziziphi spinosae semen* (Suanzaoren, fried), *Borneolum Syntheticum* (Bingpian)	Benefiting Qi and promoting blood circulation, dredging collaterals and relieving pain. For the treatment of angina pectoris and ischemic stroke	[5]<br>[13]
Shenxian Shengmai Oral Liquid	*Ginseng radix et rhizome rubra* (Hongshen), *Lycii fructus* (Gouqizi), *Salviae miltiorrhizae radix et rhizoma* (Danshen), *Epimedii folium* (Yinyanghuo), *Ephedrae herba* (Mahuang), ***Hirudo*** (Shuizhi), Psoraleae fructus (Buguzhi, processed with salt), *Asari radix et rhizoma* (Xixin)	Promoting blood circulation and removing stasis	[5]

Kangshuan Capsule	*Angelicae sinensis radix* (Danggui), *Salviae miltiorrhizae radix et rhizoma* (Danshen), *Bombyx batryticatus* (Jiangchan, fried), *Gekko japonicus Dumeril et Bibron* (Bihu), *Eupolyphaga* or *Steleophaga* (Tubeichong), *Scolopendra* (Wugong), ***Hirudo*** (Shuizhi), *Vespae nidus* (Fengfang), *Pheretima* (Dilong), *Strychni semen Praeparata* (Zhi Maqianzi), *Moschus* (Shexiang), Toad venom (Chansu, processed with wine), *Glycyrrhizae radix et rhizoma* (Gancao), *Smilacis glabrae rhizoma* (Tufuling), *Corydalis yanhusuo* (Yanhusuo, processed with vinegar), *Dryariae rhizoma Praeparata* (Zhi Gusuibu), *Zaocys* (Wushaoshe, processed with wine), *Tabanus* (Mengchong), *Squama manitis* (Chuanshanjia, processed with hot sand)	Promoting blood circulation and removing stasis. For the treatment of thromboangiitis obliterans	[14]
Mailuo Shutong Granule	*Astragali radix* (Huangqi), *Lonicerae japonicae flos* (Jinyinhua), *Phellodendri chinensis cortex* (Huangbo), *Atractylodis rhizoma* (Cangzhu), *Coicis semen* (Yiyiren), *Scrophulariae radix* (Xuanshen), *Angelicae sinensis radix* (Danggui), *Paeoniae radix alba* (Baishao), *Glycyrrhizae radix et rhizoma* (Gancao), ***Hirudo*** (Shuizhi), *Scolopendra* (Wugong), *Scorpio* (Quanxie)	Heat-clearing and detoxifying, disperse blood stasis and dredge collateral, expelling damp and removing edema. For the treatment of superficial thrombophlebitis	[14]
Zhiloushu Pill	*Rhei radix et rhizoma* (Dahuang), *Scorpio* (Quanxie), *Vespae nidus carbonisatus* (Fengfang Tan), *Bombyx batryticatus* (Jiangchan, fried), *Scolopendra* (Wugong), ***Hirudo*** (Shuizhi, fried), *Sophorae flos* (Huai hua, fried), *Pharbitidis semen* (Qianniuzi), *Crinis carbonisatus* (Xueyutan), *Eupolyphaga* or *Steleophaga* (Tubeichong, fried with wine)	Heat-clearing and expelling damp. Treating internal hemorrhoid in the early stage, mixed hemorrhoid and archosyrinx.	[5]
Shenhailong Pill, Capsule	*Syngnathus* (Hailong), *Hippocampus* (Haima), *Cervi cornu pantotrichum* (Lurong), *Cnidii fructus* (Shechuangzi), *Epimedii folium* (Yinyanghuo), *Cistanches herba* (Roucongrong), *Schisandrae Chinensis Fructus* (Wuweizi), *Ginseng Radix et Rhizoma* (Renshen), *Astragali radix* (Huangqi), *Jujubae fructus* (Dazao), *Poria* (Fuling), *Amomi fructus* (Sharen), *Dioscoreae rhizoma* (Shanyao), *Zingiberis Rhizoma* (Ganjiang),	Warmly invigorating kidney-Yang. Treating related diseases induced by kidney-Yang deficiency.	[14]

	Aconiti lateralis radix praeparata (Fuzi), *Angelicae sinensis radix* (Danggui), *Rehmanniae radix praeparata* (Shudihuang), *Asparagi radix* (Tiandong), *Ophiopogonis radix* (Maidong), *Lycii fructus* (Gouqizi), *Persicae semen* (Taoren), ***Hirudo*** (Shuizhi), *Moutan cortex* (Mudanpi), *Achyranthis bidentatae radix* (Niuxi), *Glycyrrhizae radix et rhizoma* (Gancao, processed)		
Liaoshen Capsule	Mianyeliao, ***Hirudo*** (Shuizhi), *Corydalis yanhusuo* (Yanhusuo, processed with vinegar), *Rubiae radix et rhizome* (Qiancao), *Ginseng Radix et Rhizoma* (Renshen), *Polygoni multiflori radix* (Heshouwu), *Morindae officinalis radix* (Bajitian), *Polygonati odorati rhizoma* (Yuzhu), *Scutellariae barbatae herba* (Banzhilian), *Cremastrae pseudobulbus or Pleiones pseudobulbus* (Shancigu), *Sophorae tonkinensis radix et rhizoma* (Shandougen), *Pruneliae spica* (Xiakucao), *Amomi fructus* (Sharen), *Citri sarcodactylis fructus* (Foshou), *Hordei fructus germinatus* (Maiya)	Auxiliary drugs for esophagus cancer and gastric carcinoma.	[5]
Danguixiang Granule	*Astragali radix* (Huangqi, processed), *Cinnamomi ramulus* (Guizhi), *Euodiae fructus* (Wuzhuyu) *Cinnamomi cortex* (Rougui), *Asari radix et rhizoma* (Xixin), *Persicae semen* (Taoren), *Carthami Flos* (Honghua), *Angelicae sinensis radix* (Danggui), *Chuanxiong rhizome* (Chuanxiong), *Paeoniae radix rubra* (Chishao), *Salviae miltiorrhizae radix et rhizome* (Danshen), *Moutan cortex* (Mudanpi), *Corydalis Yanhusuo* (Yanhusuo), *Wenyujin Rhizoma Concisum* (Pianjianghuang), *Sparganii rhizoma* (Sanleng), *Curcumae Rhizoma* (Ezhu), ***Hirudo*** (Shuizhi), *Aucklandiae radix* (Muxiang), *Aurantii fructus* (Zhiqiao), *Linderae radix* (Wuyao), *Coptidis rhizoma* (Huanglian), *Rehmanniae radix* (Dihuang), *Glycyrrhizae radix et rhizoma* (Gancao, processed)	Gastrosis induced by deficient cold of spleen and stomach as well as chronic atrophic gastritis	[14]<br>[13]
Zhidan Huoluo Capsule	***Hirudo*** (Shuizhi), *Salviae miltiorrhizae radix et rhizoma* (Danshen)		[5]

Gongliuqing Capsule	*Rhei radix et rhizoma* (Dahuang, processed), *Eupolyphaga* or *Steleophaga* (Tubeichong), ***Hirudo*** (Shuizhi)200g *Persicae semen* (Taoren), *Typhae pollen* (Puhuang), *Scutellariae radix* (Huangqin), *Aurantii fructus immaturus* (Zhishi), *Ostreae concha* (Muli), *Rehmanniae radix* (Dihuang), *Paeoniae radix alba* (Baishao), *Glycyrrhizae radix et rhizoma* (Gancao)	Invigorating blood, removing blood stasis. For the treatment of gynecopathy diseases induced by blood stasis	[14]<br>[13]
Ganpikang Capsule	*Bupleuri radix* (Chaihu), *Astragali radix* (Huangqi), *Citri Reticulatae Pericarpium* (Qingpi), *Paeoniae radix alba* (Baishao), *Atractylodis macrocephalae rhizoma* (Baizhu), *Isatidis radix* (Banlangen), *Curcumae longae rhizoma* (Jianghuang), *Poria* (Fuling), ***Hirudo*** (Shuizhi), *Notoginseng radix et rhizoma* (Sanqi), *Curcumae radix* (Yujin), *Galli gigerii endothelium corneum* (Jineijin, fried), Powdered *Felursi* (Xiongdan fen), Powdered *buffalo horn* extract (Shuiniujiao Nongsuofen)	Soothing Liver and strengthening Spleen, invigorating blood and clearing heat. Treating stagnation of liver-Qi with deficiency of the spleen, as well as chronic hepatitis and hepatocirrhosis in early stage	[14]
Shugan Yiyang Capsule	*Tribuli frucuts* (Jili), *Bupleuri radix* (Chaihu), *Vespae nidus* (Fengfang), *Pheretima* (Dilong), ***Hirudo*** (Shuizhi), *Aspongopus* (Jiuxiangchong), Zixiaohua, *Cnidii fructus* (Shechuangzi), *Polygalae radix* (Yuanzhi), *Cistanches herba* (Roucongrong), *Cuscutae semen* (Tusizi), *Schisandrae Chinensis Fructus* (Wuweizi), *Morindae officinalis radix* (Bajitian), *Scolopendra* (Wugong), *Acori tatarinowii rhizoma* (Shichangpu)	Functional impotence induced by liver and kidney deficiency	[5]
Naoxueshu Oral Liquid	*Astragali radix* (Huangqi), *Moutan cortex* (Mudanpi), ***Hirudo*** (Shuizhi), *Rhei radix et rhizoma* (Dahuang), *Acori tatarinowii rhizoma* (Shichangpu), *Chuanxiong rhizome* (Chuanxiong), *Achyranthis bidentatae radix* (Niuxi)	Mild and moderate hemorrhagic stroke	[5]
Tiandan Tongluo Tablet, Capsule	*Chuanxiong rhizome* (Chuanxiong), *Siegesbeckiae herba* (Xixiancao), *Salviae miltiorrhizae radix et rhizome* (Danshen), ***Hirudo*** (Shuizhi), *Gastrodiae rhizoma* (Tianma), *Sophorae flos* (Huai hua), *Acori tatarinowii rhizoma* (Shichangpu), *Bovis*	Stroke, cerebral infarction in acute stage, in early recovery	[14]

	calculus artifactus (Rengong Niuhuang), *Astragali radix* (Huangqi), *Achyranthis bidentatae radix* (Niuxi)	stage	
Qianlie Jiedu Capsule	***Hirudo*** (Shuizhi), *Rhei radix et rhizoma* (Dahuang, processed with wine), *Leonuri Herba* (Yimucao), Taraxaci herba (Pugongying), *Carthami Flos* (Honghua), *Pheretima* (Dilong), *Astragali radix* (Huangqi), *Angelicae sinensis radix* (Danggui), *Paeoniae radix alba* (Baishao), *Galli gigerii endothelium corneum* (Jineijin), *Bupleuri radix* (Chaihu)	For chronic prostatitis induced by dampness and heat as well as blood stasis	[5]
Xiaoke Tongmai oral liquid	*Astragali radix* (Huangqi), *Puerariae lobatae radix* (Gegen), *Coptidis rhizoma* (Huanglian), *Rehmanniae radix* (Dihuang), *Salviae miltiorrhizae radix et rhizome* (Danshen), *Scrophulariae radix* (Xuanshen), *Paeoniae radix alba* (Baishao), ***Hirudo*** (Shuizhi)、 *Chuanxiong rhizoma* (Chuanxiong), *Ophiopogonis radix* (Maidong), *Scutellariae radix* (Huangqin), *Cyathulae radix* (Chuanniuxi)	For type II diabetes and related complications	[5]
